# Quality of nursing care in pain management in orthopedic surgical patients: a scoping review*

**DOI:** 10.1590/1980-220X-REEUSP-2024-0110en

**Published:** 2024-12-06

**Authors:** Bárbara Ventura Fontes, Amanda Magalhaes de Oliveira, Érica Brandão de Moraes, Juliane de Macedo Antunes, Marina de Góes Salvetti, Thalita Gomes do Carmo

**Affiliations:** 1 Universidade Federal Fluminense, Escola de Enfermagem Aurora de Afonso Costa, Niterói, RJ, Brazil.; 2 Instituto Nacional de Traumatologia e Ortopedia, Rio de Janeiro, RJ, Brazil.; 3 Centro Brasileiro para o Cuidado à Saúde Informado por Evidências: Centro de Excelência do Instituto Joanna Briggs, São Paulo, SP, Brazil.; 4 Universidade de São Paulo, Escola de Enfermagem, Departamento de Enfermagem Médico-Cirúrgica. São Paulo, SP, Brazil.

**Keywords:** Trauma Nursing, Quality of Health Care, Patient Safety, Pain, Postoperative, Evidence-Based Nursing, Enfermería de Trauma, Calidad de la Atención de Salud, Seguridad del Paciente, Dolor Postoperatorio, Enfermería Basada en la Evidencia

## Abstract

**Objective:**

To map the evidence on quality nursing care practices in pain management in orthopedic surgical patients.

**Method:**

Scoping review, as per the JBI Manual recommendations. Searches were performed in the MEDLINE (PubMed), LILACS (Regional VHL), Scopus, Embase, Web of Science, Cochrane, Cinahl databases, and gray literature, regardless of language and period. Selection and extraction were performed by two independent reviewers, using inclusion/exclusion criteria, and the extracted data were organized to reflect key themes or recurring patterns related to the purpose of the review.

**Results:**

A total of 94 studies were included, most from the United States, corresponding to 34% of the sample, and published between 1997 and 2022. The findings were categorized into: nursing quality practices in pain management related to the organization and monitoring of units, and pre- and post-operative period.

**Conclusion:**

The research revealed that quality nursing care practices in pain management in orthopedic surgical patients encompass a variety of approaches, from the use of nonpharmacological practices and patient education to the use of pain assessment scales, staff training, to innovative pharmacological procedures.

## RESUMEN

**Objetivo:**

Mapear la evidencia sobre las prácticas de atención de enfermería de calidad en el manejo del dolor en pacientes de cirugía ortopédica.

**Método:**

Revisión del alcance, según las recomendaciones del Manual del JBI. Las búsquedas se realizaron en las bases de datos MEDLINE. (PubMed), LILACS (BVS Regional), Scopus, Embase, Web of Science, Cochrane, Cinahl y literatura gris, independientemente del idioma y época. La selección y extracción fueron realizadas por dos revisores independientes, utilizando criterios de inclusión/exclusión, y los datos extraídos se organizaron para reflejar temas clave o patrones recurrentes relacionados con el objetivo de la revisión.

**Resultados:**

Se incluyeron 94 estudios, la mayoría de Estados Unidos, correspondientes al 34% de la muestra, y publicados entre 1997 y 2022. Los hallazgos se categorizaron en prácticas de enfermería de calidad en el manejo del dolor relacionadas con la organización y seguimiento de las unidades, y períodos pre y postoperatorios.

**Conclusión:**

La investigación reveló que las prácticas de atención de enfermería de calidad en el manejo del dolor en pacientes de cirugía ortopédica abarcan una variedad de enfoques, desde el uso de prácticas no farmacológicas y la educación del paciente hasta el uso de escalas de evaluación del dolor, capacitación de equipos, capacitación e incluso procedimientos farmacológicos innovadores.

## INTRODUCTION

The International Association for the Study of Pain (IASP) has updated the concept of pain as an unpleasant sensory and emotional experience associated with, or resembling that associated with, actual or potential tissue damage ^(1)^. Within this new concept, important aspects must be considered: pain is always a personal experience that is influenced, to varying degrees, by biological, psychological, and social factors; pain and nociception are different phenomena, that is, pain cannot be determined exclusively by the activity of sensory neurons; through life experiences, people learn the concept of pain; a person’s report on a pain experience must be respected. Although pain often serves an adaptive role, it can have adverse effects on social and psychological function and well-being; and verbal description is just one of several behaviors for expressing pain^(1)^.

In addition to its subjective aspect, pain integrates underlying physiological processes involving the sensory and autonomic nervous systems, circulating catecholamines, and other stress response hormones, and immune system responses to autonomic and hormonal signaling. Clinically, pain states can be acute or chronic. Acute pain is characterized as a symptom and tends to resolve itself when the cause of the pain is resolved. Chronic pain, in turn, is characterized by a pathological process, with changes in the pain modulation system, and central sensitization^(2)^.

Postoperative pain is the most common form of acute pain, affecting 80% of patients undergoing surgery. The recovery time after the procedure directly impacts the frequency and intensity of pain, which tends to be more intense in the first 24 hours post-operatively^(2,3)^. It is generally a predictable and temporary consequence of the physical injury caused by the surgical procedure, a protective biological function that aids in recovery by limiting movements and behaviors that may cause additional damage to the tissue, as well as a response of the immune and inflammatory systems for the recovery of the injured tissue^(2,4)^.

Patients undergoing orthopedic surgery may experience pain in its acute, chronic form, or a combination of both, with less than half of all surgical patients reporting adequate pain relief^(5)^. In orthopedic surgeries, effective pain relief allows for better mobility, rehabilitation and accelerates the return to daily activities^(6)^.

Despite increased focus on understanding and treating postoperative pain and the development of evidence-based recommendations and best practices in the pursuit of effective pain management, postoperative pain control remains suboptimal regardless of hospital system, type of surgery, or country^(2,7)^. Inadequate pain control during the postoperative period can result in chronic pain, negatively affecting patients’ quality of life and increasing care costs for health services.^(2,3,4,8)^. Aiming at improving the quality of pain management, accrediting institutions, such as the *Joint Commission*, sought to establish standards that include quality practices in care for various professional categories, including nursing^(8)^.

The search for constant improvement in the quality of health care brings changes that generate better results for patients, integrating elements of clinical practice that can be standardized in a given context^(2)^.

In the hospital context, the nurse is the health professional who is most frequently responsible for evaluating the patient’s response to the therapy used for pain relief, as they are the ones who manage the administration of prescribed analgesia and the use of non-pharmacological practices^(9)^. Therefore, the nursing team plays a fundamental role in pain management because they are the health professionals who work on the front line in patient care. Therefore, pain assessment and care are essential skills and are fundamental to the quality of care provided to patients^(10)^.

Effective pain management is the main objective of postoperative care and contributes to patient recovery, return to activities, comfort and a sense of well-being and satisfaction, and reduction of time, and costs of hospitalization^(11)^. Although there is consensus among scientific evidence and health professionals about the importance of quality pain management in the postoperative period, pain control after a surgical procedure remains a major problem during hospitalization, with pain reported as moderate to severe in 51% of patients^(7)^.

Identifying quality practices in pain management contributes to the search for high-quality, safe and efficient care, benefiting both patients and professionals, and is essential to advancing knowledge in the area and improving health care^(2,10)^.

Some primary studies have been published on the topic and a preliminary search of the Medical Literature Analysis and Retrieval System Online (MEDLINE), COCHRANE DATABASE and JBI Evidence Synthesis databases was conducted, and no systematic or scoping reviews on the topic were found. The objective of this study was to map the evidence on quality nursing care practices in pain management in orthopedic surgical patients.

## METHOD

### Design of Study

This is a scoping review study based on the theoretical framework proposed and developed by the JBI, specific to this type of study^(12)^, being reported according to the assumptions of the *Preferred Reporting Items for Systematic reviews and Meta-Analyses extension or Scoping Reviews* (PRISMA-ScR)^(13)^.

The scoping review is well suited to this research, which aims to take a broad approach to the topic to find gaps in the literature and support future research. The protocol for this review was registered, as recommended by JBI, on the registration platform *Open Science Framework* (https://osf.io/tfwuh) with DOI 10.17605/OSF.IO/TFWUH^(14)^.

### Review Question Identification

To construct the question, the acronym PCC (*Population, Concept* and *Context*), recommended for scoping reviews, was used. Based on these definitions, the research question was established: “Which quality nursing care practices in pain management in orthopedic surgical patients are recommended and performed in the hospital setting?”, where Population = Orthopedic surgical patients; Concept = Quality nursing practices in pain management; and Context = hospital environment.

### Eligibility Criteria

Population: Studies involving adult orthopedic surgical patients in a hospital setting during the pre- and/or post-operative period were considered, regardless of the nature or extent of the surgery performed. Studies that did not answer the research question were excluded.

Concept: As a concept, studies containing quality nursing care practices in pain management in orthopedic surgical procedures were considered. The quality of nursing care is defined as all practices that involve holistic patient care, which encompasses issues of meeting patient needs, nurses’ competence and empathy, better patient outcomes and satisfaction^(15)^. For pain management, studies were considered that described practices related to the essential competencies of nurses in the management of acute and chronic pain, which involve the multidimensional nature of pain, pain assessment and measurement, pain management, and the context of pain management^(16)^.

Context: The research context was developed in the hospital environment, where medical care and patient treatment activities are concentrated. This environment is crucial for understanding pain management during the surgical period and issues related to hospital management and health policies.

Design of study: We considered experimental and quasi- experimental study designs, including randomized and non-randomized clinical trials, before-and-after studies, and time series. Observational studies, including cohort studies, case- control studies and cross-sectional studies, case series and case reports, as well as clinical practice protocols and guidelines. Literature reviews, dissertations and text articles, and expert opinion were also considered for inclusion in this scoping review.

Publications that did not meet the objectives of the study, that did not contain information relevant to the proposed scenario, and that did not address the chosen concept and context were excluded.

### Search Strategy and Information Sources

The search took place from May to October 2023, by two independent reviewers, and a three-stage search strategy was used for this review. An initial limited search of MEDLINE (Pubmed) and CINAHL was performed, followed by analysis of the text words contained in the title and abstract, and the index terms used to describe the article, as exemplified in Chart 1. A thorough secondary search was conducted across all databases included in the review: MEDLINE (PubMed), LILACS (Regional VHL), Scopus, Embase, Web of Science, Cochrane and Cinahl, using the key words and index terms identified in the initial search. To help identify any additional studies, a tertiary literature search was conducted by examining the reference lists of all literature that met the inclusion criteria for this review. The review considered studies in any language and with no publication date limit. The grey literature search included: Websites of pain organizations, Digital Library of Theses and Dissertations, Protocols and Clinical Guidelines recognized by government agencies and *National Institute for Health and Care Excellence* (NICE).

**Chart 1:** Search and mapping of terms carried out in the MEDLINE database on 05/22/2023 Niterói, RJ, Brasil, 2023.

Search strategy		Result
#1	"Orthopedic Procedures" [Mesh] OR"Orthopedic Procedure" OR "Procedure, Orthopedic" OR "Procedures, Orthopedic" OR "Orthopedic Surgical Procedures" OR "Orthopedic Surgical Procedure" OR "Procedure, Orthopedic Surgical" OR "Procedures, Orthopedic Surgical" OR "Surgical Procedure, Orthopedic" OR "Surgical Procedures, Orthopedic" OR "Orthopedic Sur-gery" OR "Orthopedic Surgeries" OR "Surgeries, Orthopedic" OR "Surgery, Orthopedic" OR "Orthopedic Rehabilitation Surgery" OR "Orthopedic Rehabilitation Surgeries" OR "Rehabili-tation Surgeries, Orthopedic" OR "Rehabilitation Surgery, Orthopedic" OR "Surgeries, Or-thopedic Rehabilitation" OR"Surgery, Orthopedic Rehabilitation"	432.000
#2	("Pain, Postoperative"[Mesh] OR "Post-surgical Pain"[tiab] OR "Post surgical Pain"[tiab] OR"Postsurgical Pain"[tiab] OR "Post operative Pain"[tiab] OR "Post operative Pain"[tiab] OR "Post-operative Pains"[tiab] OR "Postoperative Pain"[tiab] OR "Chronic Postoperative Pain"[tiab] OR "Chronic Post-surgical Pain"[tiab] OR "Chronic Post surgical Pain"[tiab] OR "Chronic Postsurgical Pain"[tiab] OR "Persistent Postsurgical Pain"[tiab] OR "Chronic Post operative Pain"[tiab] OR "Chronic Post operative Pain"[tiab] OR "Acute Postoperative Pain"[tiab] OR"Acute Post-operative Pain"[tiab] OR "Acute Post operative Pain"[tiab])	62,084
#3	"Orthopedic Nursing"[Mesh] OR "Nursing, Orthopedic"	1,157
#4	#1 AND #2 AND #3	24

### Study Selection

Records were imported into the EndNote reference manager (Clarivate Analytics, PA, USA) and duplicate studies were removed. Then, the generated EndNote references were exported to the online platform for systematic reviews Rayyan QCRI^(17)^. Subsequently, the studies were selected in two stages by two independent reviewers. First, through the analysis of titles and abstracts and, then, the selected studies that met the eligibility criteria and that had consensus between the two reviewers were read in full for inclusion or exclusion. Any disagreements arising between reviewers were resolved through discussion, and there was no need for a third reviewer.

### Data Extraction and Presentation of Results

The information from the documents selected for analysis was independently extracted by two reviewers, using spread­sheets from Microsoft Excel®. The extracted data included specific details about the population, concept, context, study methods, and main findings relevant to the purpose of the review. The first part of the extraction profiled the studies containing the following topics: Author and year of publication; Study location; Design of Study; Sample size; Type of orthopedic surgery and Hospital (type and hospitalization sector). Following data extraction, the findings were consolidated in a table that highlighted the main characteristics of the studies included in the research, providing an overview of all the material used and its relevance to the topic, and were described regarding: quality nursing practices in pain management related to the organization of units and patient monitoring; quality nursing practices in pain management in the pre- and postoperative period. Therefore, the data extracted were organized to reflect key themes or recurring patterns related to the purpose of the review.

Data are presented in figures and tables, accompanied by a narrative analysis.

## RESULTS

**Figure 1:**
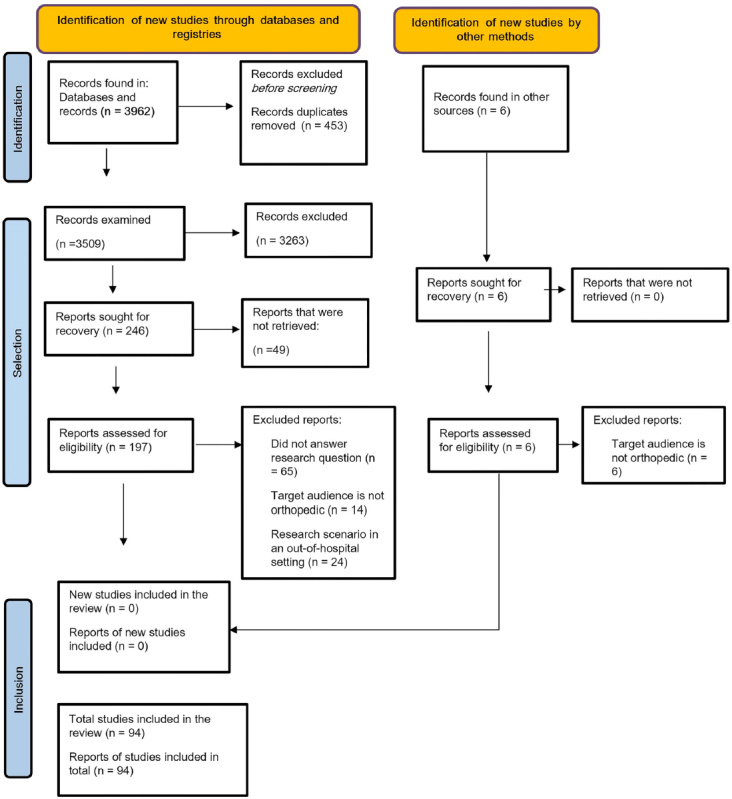
PRISMA-ScR flowchart for publication selection^(13)^ – Niterói, RJ, Brasil, 2023.

The database search retrieved 3,962 potentially relevant studies/records. A total of 453 duplicate documents were excluded. The title and abstract of 3,509 publications were analyzed, with 3,263 being excluded for not meeting the inclusion criteria. Thus, 246 studies were fully assessed for eligibility. At the end, 94 studies^(18,19,20,21,22,23,24,25,26,27,28,29,30,31,32,33,34,35,36,37,38,39,40,41,42,43,44,45,46,47,48,49,50,51,52,53,54,55,56,57,58,59,60,61,62,63,64,65,66,67,68,69,70,71,72,73,74,75,76,77,78,79,80,81,82,83,84,85,86,87,88,89,90,91,92,93,94,95,96,97,98,99,100,101,102,103,104,105,106,107,108,109,110,111)^ were included to compose the final review sample (Figure 1).

Chart 2 presents a summary of the studies. Of the 94 studies included, 90 were published in English^(18,19,20,21,23,24,25,26,27,28,29,30,31,32,33,34,35,38,39,40,41,42,43,44,45,46,47,48,49,50,51,52,53,54,55,56,57,58,59,60,61,62,63,64,65,66,67,68,69,70,71,72,73,74,75,76,78,79,80,81,82,83,84,85,86,87,88,89,90,91,92,93,94,95,96,97,98,99,100,101,102,103,104,105,106,107,108,109,110,111)^, two in Portuguese^(36,37)^, one in German^(22)^, and one in Polish^(77)^. Regarding the publication period, the studies are between the years 1997 and 2022, with greater growth in production from 2015 onwards. As for the origin, thirty-six were produced on the American continent^(18,19,20,21,23,24,25,30,34,36,37,40,41,42,45,46,50,51,58,59,60,61,63,68,69,71,72,74,76,78,91,92,97,98,104,108,109,110)^, twenty-nine on the Asian continent^(11,12,15,16,17,22,23,27,28,36,37,39,40,41,50,54,57,64,71,77,78,79,86)^, twenty four in Europe^(22,26,29,35,62,64,67,75,77,83,84,85,86,88,89,90,91,99,100,101,103,105,106,107,111)^, and five in the Oceania^(48,49,66,81,82)^. The countries with the highest volume of publications were: United States of America with thirty-two (34%), China with fifteen (16%), and Sweden with nine (8.5%). Among the publications analyzed, review studies stood out as the most used, comprising 35.7% of the total, followed by randomized clinical trials (25.7%). In the search in other data sources, what was retrieved was not included for not meeting the inclusion criteria; in the database search, two recommendations^(78,91)^, one consensus^(47)^, and a master’s thesis^(20)^ were included.

**Chart 2:** Profile of selected studies - Niterói, RJ, Brasil, 2023.

Article	Author/year/country	Type of surgery	Design of study	Sample	Local
A1^(18)^	Roberts et al., 2019 Estados Unidos	Knee arthroplasty	Literature Review	n = 236	Hospitalization, elective surgery
A2^(19)^	Smith-Miller et al., 2009 Estados Unidos	First time elective hip/knee surgery	Retrospective chart reviews	n = 65	Hospitalization, elective surgery
A3^(20)^	Bell, 1999 Estados Unidos	Hip and knee	Master’s thesis	n = 133	Hospitalization, elective surgery
A4^(21)^	Briggs and Closs, 1999 Estados Unidos	Different types of orthopedic surgeries	Cross-sectional descriptive	n = 417	Orthopedic, trauma and elective surgery ward
A5^(22)^	Ettrich et al., 2007 Alemanha	Different types of orthopedic surgeries	Literature Review	Does not report	Emergency and elective surgery
A6^(23)^	Pizzi et al., 2020 Estados Unidos	Hip arthroplasty	Randomized Clinical Trial	n = 30	University hospital, orthopedic unit
A7^(24)^	Wilson et al., 2016 Canadá	Knee arthroplasty	Randomized Clinical Trial	n = 143	Hospitalization, elective surgery
A8^(25)^	Laframboise-Otto et al., 2021 Estados Unidos	Knee and hip arthroplasty	Randomized Clinical Trial	n = 50	University hospital, orthopedic unit
A9^(26)^	Bell, 2000 Irlanda	Different types of orthopedic surgeries	Literature review	n = 56	Not applicable
A10^(27)^	Cho et al., 2015 Coreia do Sul	Spine surgery	Systematic review	n = 5	Hospitalization, elective surgery
A11^(28)^	Shin et al., 2019 Coreia do Sul	Hip arthroplasty	Systematic Review	n = 24	Not applicable
A12^(29)^	Moizo et al., 2003 Itália	Different types of orthopedic surgeries	Observational prospective	n = 592	Surgical care hospital
A13^(30)^	Titler et al., 2003 Estados Unidos	Hip arthroplasty	Experimental	n = 709	Intensive Care Hospital
A14^(31)^	Lin, 2012 Taiwan	Knee and hip arthroplasty	Case Study control	n = 93	Medical Center
A15^(32)^	Yu et al., 2021 China	Trauma	Case-control study	n = 78	Orthopedic trauma emergency
A16^(33)^	Xie et al., 2022 China	Spine	Randomized Clinical Trial	n = 126	General hospital; elective surgery
A17^(34)^	Adeboye et al., 2021 Estados Unidos	Knee arthroplasty/Spinal arthrodesis	Cohort study	n = 49	Emergency
A18^(35)^	Milutinović et al., 2009 Sérvia	Genital Orthoplastic Surgery; Thigh Orthoplastic Surgery, Ligamentoplasty	Cross-sectional study	n = 135	Institute of Public Health
A19^(36)^	Paula et al., 2011 Brasil	Hip, femur, knee	Literature review	n = 18	General Hospital and PACU
A20^(37)^	Barbosa, 2014 Brasil	Elective surgeries, except head, neck, neurological and those undergoing local anesthesia.	Prospective study	n = 132	University Hospital
A21^(38)^	Wang et al., 2015 China	Knee arthroplasty	Quasi-experimental design	n = 66	General Hospital
A22^(39)^	Wong et al., 2014 China	Trauma: hip or femur	Quasi-experimental design	n = 150	General Hospital; Ward
A23^(40)^	Petrie and Matzkin, 2019 Estados Unidos	Knee and hip arthroplasty	Literature Review	n = 10	Hospital
A24^(41)^	Harper et al., 2015 Estados Unidos	Knee and hip arthroplasty	Randomized Clinical Trial	n = 72	Outsourced hospital
A25^(42)^	Huang et al., 2001 Estados Unidos	Different types of orthopedic surgeries	Literature Review	n = 39	General hospital; Ward and Outpatient Clinic
A26^(43)^	Myoji et al., 2015 Japão	Hip arthroplasty	Case-control study	n = 34	General Hospital
A27^(44)^	Wang et al., 2012 China	Different types of orthopedic surgeries	Cross-sectional study	n = 1131	General hospital; Level II and III ward
A28^(45)^	Abou-Setta et al., 2011 Canadá	Hip fracture	Systematic Review	83 studies	General Hospital; Ward
A29^(46)^	DeWaters et al., 2008 Estados Unidos	Knee and hip arthroplasty	Descriptive correlational design.	n = 25	General hospital; Orthopedics
A30^(47)^	Wainwright et al., 2020 Inglaterra	Knee and hip arthroplasty	Consensus	17 Research Topics	General Hospital; Ward
A31^(48)^	Adie et al., 2012 Austrália	Knee arthroplasty	Systematic Review	12 studies: n = 809	General Hospital; Ward
A32^(49)^	Botti et al., 2014 Austrália	Knee and hip arthroplasty	Multicenter study	n = 374	General Hospital; Ward
A33^(50)^	Weekes et al., 2019 Estados Unidos	Arthroscopic rotator cuff repair	Randomized clinical trial	n = 151	General Hospital; Ward
A34^(51)^	Wittig-Wells et al., 2015 Estados Unidos	Knee arthroplasty	Randomized clinical trial	n = 29	Orthopedic Hospital
A35^(52)^	Xue et al., 2018 China	Unilateral elbow arthroplasty	Randomized clinical trial	n = 60	University hospital; Ward
A36^(53)^	Patiyal et al., 2021 Indonésia	Knee arthroplasty	Systematic Review	n = 778	General Hospital; Ward
A37^(54)^	Seers et al., 2008 Inglaterra	Knee and hip arthroplasty	Randomized Clinical Trial	n= 118	Orthopedic Hospital; Ward
A38^(55)^	Ko et al., 2021 China	Knee arthroplasty	Systematic Review	n = 891	General Hospital; Ward
A39^(56)^	Qi et al., 2017 China	Hip arthroplasty	Case-control study	n = 100	Surgical department
A40^(57)^	Rucinski, K., 2020 Estados Unidos	Knee and hip arthroplasty	Systematic Review	n = 778	General Hospital; Ward
A41^(58)^	Duncan and Pozehl, 2000 India	Knee arthroplasty	Quasi-experimental	n = 240	General Hospital; Ward
A42^(59)^	Lim et al., 2014 Singapura	Knee arthroplasty	Quasi-experimental	n = 18	General Hospital; Ward
A43^(60)^	Schroeder et al., 2016 Estados Unidos	Knee arthroplasty	Single design, pre/post intervention	n = 30 nurses n = 100 patients	General Hospital; Orthopedics Unit
A44^(61)^	See et al., 2017 Singapura	Knee and hip arthroplasty	Systematic review	20 articles	General Hospital; Ward
A45^(62)^	Yılmazer, 2017 Turquia	Different types of orthopedic surgeries	Descriptive	n = 63 nurses n = 315 patients	Orthopedic Clinic; Hospitalization
A46^(63)^	Carpenter et al., 2017 Estados Unidos	Different types of orthopedic surgeries	Integrative Review	9 articles	General Hospital; Ward
A47^(64)^	Wikström et al., 2016 Suécia	Different types of orthopedic surgeries	Exploratory design	n = 24	General Hospital and Orthopedics Unit
A48^(65)^	Keast et al., 2022 Austrália	Knee arthroplasty	Qualitative exploratory-descriptive	n = 120	General Hospital; Ward
A49^(66)^	Cui et al., 2018 China	Elective orthopedic joint surgery,	Separate interventional sample, pre- and post-test	n = 77	General Hospital; Ward
A50^(67)^	Karlsten et al., 2005 Suécia	Different types of orthopedic surgeries	Descriptive qualitative study	n = 270	University Hospital; general surgery and orthopedics
A51^(68)^	Neitzel et al., 1999 Estados Unidos	Knee and hip arthroplasty	Literature Review - Evidence-Based Practice	n = 118	General Hospital; Ward
A52^(69)^	Doi et al., 2014 Estados Unidos	Knee and hip arthroplasty	Literature Review - Evidence-Based Practice	n = 63	General Hospital; Ward
A53^(70)^	Huang et al., 2022 China	Knee arthroplasty	Randomized Clinical Trial	n = 82	General Hospital; Ward
A54^(71)^	Colwell Jr, 2008 Estados Unidos	Knee arthroplasty	Literature Review	n = 50	General Hospital; Ward
A55^(72)^	Schinsky et al., 2016, Estados Unidos	Knee arthroplasty	Prospective study	n = 100	General Hospital; Ward
A56^(73)^	Wang and Tian, 2021 China	Hip replacement surgery	Quasi-experimental	n = 38	Orthopedic Hospital; Ward and Trauma Center
A57^(74)^	Brown Jr, 2008 Estados Unidos	Shoulder arthroplasties	Literature review	n = 24	Not applicable
A58^(75)^	Specht et al., 2015 Dinamarca	Knee and hip arthroplasty	Retrospective and descriptive design	n = 310	General Hospital
A59^(76)^	Hardwick et al., 2012 Estados Unidos	Knee arthroplasty	Randomized Clinical Trial	n = 41	General Hospital
A60^(77)^	Skokowska et al., 2016 Polônia	Hip arthroplasty and arthroscopy	Evidence-based practical literature review.	n = 112	Emergency room and orthopedics
A61^(78)^	Nussenzveig, 1999 Estados Unidos	Knee and hip arthroplasty	Experience Report	20 medical records	Post-operative wards
A62^(79)^	Colquhoun et al., 2019 Escócia	Amputation	Implementation	-	Professionals linked to NHS Tayside Pain Service
A63^(80)^	Wang et al., 2020 China	Fracture treatment following trauma	Case-control	n = 220	Trauma Post-operative period
A64^(81)^	McDonall et al., 2019 Austrália	Knee arthroplasty	Cluster-randomized, four-period crossover, and concurrent process evaluation	n = 241	General Hospital; Ward
A65^(82)^	McDonall et al., 2016 Austrália	Knee arthroplasty	Crossover trial, simultaneous and randomized process.	n = 240	Orthopedic wards
A66^(83)^	Sjöling et al., 1997 Suécia	Knee and hip arthroplasty	Implementation	n = 59	Post-operative wards
A67^(84)^	Niemi-Murola et al., 2007 Finlandia	Knee and hip arthroplasty	Descriptive	n = 77 patients n = 63 nurses	Post-operative wards
A68^(85)^	Stomberg and Oman, 2006 Suécia	Hip arthroplasty	Descriptive and comparative	n = 112	Post-operative wards
A69^(86)^	Joelsson et al., 2009 Suécia	Hip arthroplasty	Descriptive	n = 50	Orthopedic ward
A70^(87)^	Zhu et al., 2019 China	Knee and hip arthroplasty	Qualitative	n = 45	Post-op orthopedic surgery ward
A71^(88)^	Ingadottir et al., 2016 Islândia	Not specified	Qualitative descriptive	n = 13	University hospital
A72^(89)^	Yıldırım et al., 2015 Turquia	Not specified	Quantitative, descriptive, and comparative study	n = 350	Orthopedic Clinics
A73^(90)^	Idvall et al., 2008 Suécia	Not specified	The qualitative and descriptive approach using individual patient interviews	n = 30	Orthopedic and general surgery wards
A74^(91)^	Arkin et al., 2022 Estados Unidos	Orthopedic surgery	Recommendation of a society of experts	n = 30	Post-operative care
A75^(92)^	Notte et al., 2016 Estados Unidos	Knee arthroplasty	Pilot study	n = 43	Post-operative care
A76^(93)^	Wong et al., 2010 China	Trauma - Limb Fracture	Quasi-experimental	n = 125	General Hospital; Ward
A77^(94)^	Chen et al., 2013 China	Knee arthroplasty	Quasi-experimental	n = 92	Orthopedic Hospital Ward
A78^(95)^	Rahmani et al., 2019 Iran	Knee arthroplasty	Non-randomized clinical trial with control group	n = 46	Orthopedic Hospital Ward
A79^(96)^	Pasyar et al., 2018 Iran	Trauma - Limb Fracture	Randomized clinical trial	n = 66	Orthopedic Hospital Ward
A80^(97)^	Schneider, 2016 Estados Unidos	Knee arthroplasty	Descriptive, comparative and quasi-experimental.	n = 65	Acute hospital unit that includes a population of orthopedic patients
A81^(98)^	McCaffrey and Locsin, 2006 Estados Unidos	Hip/Knee surgeries	Randomized controlled clinical trial.	Experimental group n = 62 Control Group n = 62	Orthopedic ward
A82^(99)^	Büyükyılmaz and Aştı, 2013 Turquia	Knee and hip arthroplasty	Experimental design	n = 60	Orthopedic Hospital; Orthopedic and Trauma ward
A83^(100)^	Erden et al., 2016 Turquia	Arthroscopic shoulder surgery.	Experimental design	n = 101	Day hospital
A84^(101)^	Elmali and Balci Akpinar, 2016 Turquia	Not specified	Experimental design	n = 90	Orthopedic and trauma ward
A85^(102)^	Fang et al., 2010 Taiwan	Arthroscopy	Prospective, double-blind, quasi-experimental study.	n = 59	General Hospital
A86^(103)^	Bahçelia and Karabulut, 2020 Turquia	Lumbar microdiscectomy	Experimental study with control group and randomized clinical trial design.	n = 97	Post-operative ward
A87^(104)^	Gatewood et al., 2016 Estados Unidos	Knee arthroplasty	Systematic review	n = 37	Post-operative care
A88^(105)^	Angelini et al., 2021 Suécia	Elective lumbar spine surgery	Implementation	n = 81	Ward
A89^(106)^	Sjo ¨linga and Nordahl, 2002 Suécia	Knee arthroplasty	Prospective experimental design with two parallel groups	n = 60	Orthopedic unit
A90^(107)^	Andersson et al., 2015 Suécia	Knee and hip arthroplasty	Descriptive qualitative study	n = 18	Orthopedic unit
A91^(108)^	Lambert and Cata, 2014 Estados Unidos	Knee and hip arthroplasty	Quantitative comparative study.	n = 30	Orthopedic unit
A92^(109)^	Antall and Kresevic, 2004 Estados Unidos	Knee and hip arthroplasty	Experimental design	n = 13	Orthopedic unit
A93^(110)^	Pellino et al., 2005 Estados Unidos	Knee and hip arthroplasty	Descriptive, comparative, and correlational design.	65 medical records	Orthopedic unit
A94^(111)^	Cina-Tschumi, 2007 Alemanha	Knee/Hip/Spine/ Shoulder	Literature review	17 articles	General Hospital; Ward

The quality practices in pain management found in the studies were classified according to the organization of units and patient monitoring and the operative period in which they were applied (Chart 3). Regarding the organization of units and patient monitoring, the searches used the nursing team training and evidence-based practice, the use of protocols and care planning as intervention; they were carried out regardless of the operative time and had as objective the management of pain based on the organization of services.

**Chart 3:** Nursing quality practices in pain management - Niterói, RJ, Brasil, 2023.

Nursing Quality Practices in Pain Management	
**Organization of units and patient monitoring**	Education for pain self-management^(24,25)^; Use of pain assessment scales ^(30)^; Individualized care: Manage sleep quality: ^(26,29)^; Patient participation in care decisions ^(23, 55)^; Pharmacological/new procedures: Patient-controlled analgesia^(23)^; Specialized team: Nursing team specialized in pain ^(56,82,89,103,105,109)^; Multidisciplinary team ^(35,44,59,62,105)^; Quality nursing ^(32,74,75)^; Management: Staff training ^(36,37,47,52, 55,56,57,60,62,67,69,70,73,84,87,88, 89,91,92,103,109)^; Standard protocol and education ^(19,22,26,29,36,38,41,42,44,45,63,65, 97,100,107)^; Evidence-based practice ^(29,48,49,50,65,66,67,75,80,87,103,106,110)^; Care planning ^(22,26,32,85,89,104)^; Use of algorithm ^(34,48)^; Assessment through audits ^(64,89)^; Individual performance feedback ^(58)^; Working conditions in pain assessment: ^(77)^; Flowchart ^(79)^; Use of checklist ^(105)^; Provision of non-pharmacological treatment kit ^(110)^; Creation of specific guide for cryotherapy ^(111)^;
**Preoperative period**	Use of non-pharmacological practices for pain relief: Acupuncture ^(60)^; Music therapy ^(25,53)^; Relaxation therapy (guided imagery and meditation) ^(31,54,63)^; Deep breathing ^(39,60)^; Cryotherapy ^(44)^; Transcutaneous electrical neurostimulation ^(45)^; Reiki therapy ^(92)^; Education for pain self-management ^(22,24,32,33,39,42,44,47,50,56,57,61,71,83,85, 87,89,91,93,94,95,106)^; Use of pain assessment scales ^(18,21,37,43,95,56,69,75,85)^; Individualized care: Manage sleep quality ^(43)^; Patient participation in care decisions ^(33,57,68)^; Pharmacological/new procedures: Multimodal drug therapy ^(56)^; Management: Assistance ^(37)^;
**Postoperative period**	Use of non-pharmacological practices for pain relief: Cryotherapy ^(48,50,51,52,60,62,69,72,102,104,110,111)^; Music therapy ^(25,36,53,60,73,97,98,99)^; Relaxation therapy (guided imagery and meditation) ^(22,31,36,38,54,63,69,109)^; Acupuncture ^(22,24,27 ,28,56)^; Massage ^(24, 62, 96, 97,110)^; Repositioning and early ambulation ^(33,62,69, 100)^; Deep breathing ^(39,99)^; Hyperthermotherapy ^(46, 83)^; Vibrational therapy ^(36)^; Aromatherapy^(36)^; Dog Therapy ^(41)^; Transcutaneous Electrical Neurostimulation^(45)^;Spiritual Strengthening ^(60)^; Low-Level Laser Acupuncture^(70)^; Healing Touch (HT) ^(76)^; Reiki Therapy^(92)^; Cognitive-behavioral distraction technique (Watching funny videos) ^(101)^;Education for pain self-management ^(20, 44, 57, 59,61,68,74,75,82, 87, 106)^; Use of booklet ^(95,107)^ Cognitive-behavioral approach ^(93)^; Use of pain assessment scales ^(18,21,23,34,42,43,44,46,49,56, 64,66,68,78,79, 81,91,95,100)^;
	Patient participation in care decisions ^(56,57,65,81,82,85,91,106)^; Pharmacological/new procedures: Multimodal drug therapy ^(19,29,37, 45,47,69,71)^; Patient-controlled analgesia ^(67,71,74,75,78,108)^; Pre-analgesia in the post-anesthesia care unit ^(23)^; Management: Individual performance feedback ^(58)^;

Regarding the period in which the best practices were used, we observed that the interventions studied were sometimes repeated, such as the use of some non-pharmacological practices; therefore, another classification was made in relation to the operative period (pre- and post-operative).

Data extraction revealed a series of relevant practices associated with pain management, among which the highlight was the use of non-pharmacological approaches for pain relief, evidenced in 34.4% of cases. Furthermore, cases of effective management (14.6%), education for pain self-management (13.5%), adoption of innovative procedures (10.4%), formation of specialized pain management teams (10.4%), use of pain assessment scales (5.2%), individualized attention (4.2%), and active patient participation in care decisions (7.3%) were identified.

## DISCUSSION

This review allowed the mapping of quality nursing care practices in pain management in orthopedic surgical patients. Quality of care is an essential element of healthcare delivery, and nurses play a vital role in managing postoperative pain care, ensuring that patients receive adequate and individualized pain relief, promoting their overall well-being and recovery.

The IASP also defines a minimum curriculum that brings together knowledge that nurses must have to adequately manage the pain^(112)^. In Brazil, the role of nurses in pain management is regulated by Cofen Resolution 581/2018^(113)^.

Studies mention the importance of a nursing team specialized in pain^(56,82,89,103,105,109)^, as well as the importance of multidisciplinary^(35,44,59,62,105)^ teamwork. Since pain control is an important outcome to be monitored when we think about the quality and safety of care, and that, despite this, hospitalized patients still have their pain treated ineffectively, research that focuses on strategies that can impact the pain management performed is essential to transform the practice^(9,113,114)^.

A considerable number of the selected studies was focused on professional training, evidence-based practice, definition of care protocols, and use of audits to evaluate the practice used as a form of intervention to make pain management performed by nurses effective, safe and based on best practices.

The organization of units and monitoring of the patient after surgery are crucial to ensure treatment effectiveness and safety. This includes several management practices found in the studies, such as: staff empowerment^(36,37,47,52,55,56,57,60,62,67,69,70,73,84,87,88,89,91,92,103,109)^, standard protocol implementation and education^(19,22,26,29,36,38,41,42,44,45,63,65,97,100,107)^, evidence-based practice^(30,49,50,51,66,67,68,76,80,88,104,107,111)^, care planning^(23,27,33,86,90,104)^, use of algorithm^(34,48)^, assessment through audits^(64,89)^, working conditions in pain assessment^(77)^, flowchart^(79)^, use of checklist^(105)^, provision of non-pharmacological treatment kit^(110)^, and creation of a specific guide for cryotherapy^(111)^. The aim of these strategies is to encourage a systematic and efficient approach to pain management after surgery, ensuring quality of care and continuous improvement of clinical processes.

It is important that all quality practices are based on scientific evidence, and that an institutionalized protocol is established, standardizing conduct and flows in care. Pain assessment, using validated and standardized instruments at the institution, was cited in several studies^(18,21,23,34,37,42,43,44,46,49,56,63,66,68,69,75,78,79,81,91,95,100)^. The assessment and delivery of effective and safe care is reflected in a culture of excellence and results in the achievement of aimed results^(115,116)^. One of the authors presented a different recommendation from those reported, which is individual performance feedback, a process in which health professionals receive specific information about the quality and effectiveness of their practices related to pain control in patients, and the results indicated that supplying feedback to nurses about their previous pain management practices can improve postoperative pain outcomes for patients^(58)^.

During the pre- and post-operative period, studies showed the relevance of a multimodal pain treatment, involving pharmacological and non-pharmacological approaches in pain management. This approach can minimize the amount of opioids administered and associated risks. Despite this, many surgical patients still receive only pharmacological treatment for pain. Within this context, nurses play a fundamental role in implementing non-pharmacological practices for pain management in health services. In the preoperative period, several non-pharmacological practices were employed in the studies, including acupuncture^(23)^, music therapy^(25,53,57)^, relaxation therapy (such as guided imagery and meditation)^(31,54,63)^, deep breathing^(39,60)^, cryotherapy^(45)^, transcutaneous neurostimulation^(45)^, and reiki therapy^(92)^. In the postoperative period, the most commonly used pain relief therapies included cryotherapy^(48,50,51,52,60,62,69,72,102,104,110,111)^, music therapy^(25,36,53,57,60,73,97,98,99)^, relaxation therapy (such as guided imagery and meditation)^(22,31,36,38,54,63,69,109)^, acupuncture^(22,24,27,28,55)^, massage^(24,62,96,99,110)^, repositioning and early ambulation^(33,62,69,100)^, deep breathing^(39,99)^, hyperthermotherapy^(46,83)^, vibrational therapy^(20)^, aromatherapy^(36)^, dog therapy^(41)^, transcutaneous electrical neurostimulation^(45)^, spiritual strengthening^(60)^, low-intensity laser acupuncture^(70)^, healing touch (HT)^(76)^, and Reiki therapy^(92)^.

Studies also emphasized the importance of patient-centered care. This is a model of care that places the patient at the center of the decision-making process, considering their individual needs, desires and values when planning their care ^(117)^. This model, in addition to strengthening safety in care processes, has an impact on pain management, given the multidimensionality and subjectivity of pain. Thus, education for pain self-management was cited in several studies highlighting its importance^(22,24,32,33,39,42,44,47,50,56,62,71,83,85,87,89,91,93,94,95,106)^, even as an educational policy of the institution for self-control of pain^(20,44,59,61,68,74,75,82,87,106)^.

Other studies brought individualized care with an emphasis on patients’ active participation in care decisions^(32,56,65,68,81,82,85,91,106)^ and in sleep quality management ^(39,40,41,43)^, including the use of booklets^(95,107)^ and the cognitive-behavioral approach^(101)^ as strategies. These practices aim to optimize preoperative pain management and improve the patient experience during the orthopedic surgical process.

Some studies have shown that the pain mechanism is not the same, it occurs differently for each person, being considered a personal and non-transferable^(118,119,120)^ experience, a multidimensional and subjective phenomenon, which makes its evaluation a complex process, since it cannot be objectively measured^(120)^. Thus, the studies selected in this review address precisely education for pain self-management, patient participation in care decisions and individualized care, team training and patient assessment and monitoring, highlighting the role of nurses in pain management, in the sense that when they perform well-structured and individualized pain management, the results for pain control and patient satisfaction are positive.

There are new pharmacological procedures, such as multimodal pharmacological therapy^(19,29,37,45,47,69,71)^, patient-controlled analgesia^(67,71,74,75,78,108)^, and pre-analgesia in the post-anesthesia recovery unit^(23)^. The use of pain assessment scales is common to monitor and quantify patients’ pain, providing an objective basis for treatment^(18,21,37,43,56,69,75,85,95)^. These multidimensional approaches aim to improve postoperative pain management and provide better recovery for patients.

The limitation of this study, as it is a scoping review, is that it did not assess the quality and risk of bias of the primary studies and, therefore, does not provide recommendations on the effectiveness of the practices mentioned for pain management. However, the study provides an overview of the practices highlighted in the literature, placing nurses as protagonists of quality practices in pain management in orthopedic surgical patients. This study also contributes to the strengthening of non-pharmacological practices as it brings several possibilities of use for surgical patients.

## CONCLUSION

The results of this review highlight the complexity and importance of pain management in patients undergoing orthopedic surgery. Postoperative pain was considered a significant challenge that affects these patients’ well-being and recovery. By analyzing a variety of sources, including experimental and observational studies, protocols, clinical guidelines, and gray literature, we were able to gather a wide range of valuable information. Quality practices in pain management performed by nurses were classified into organization of units and patient monitoring, and during pre- and post-operative period, bringing important actions, such as definition of protocols, team training, use of pain assessment instruments, non-pharmacological interventions and assessment through audits, which can assist leaders and professionals in the practice in patient care.

In conclusion, this research review provides a comprehensive overview of best nursing practices in the management of postoperative pain in orthopedic patients. The results and recommendations obtained can form a solid basis for improving treatment practices, improving patient outcomes and promoting continued development of treatment in this special group.

## References

[1] Raja SN, Carr DB, Cohen M, Finnerup NB, Finnerup NB, Flor H (2020). The revised International Association for the Study of Pain definition of pain: concepts, challenges, and compromises. Pain.

[2] Meissner W, Huygen F, Neugebauer E, Osterbrink J, Benhamou D, Betteridge N (2018). Management of acute pain in the postoperative setting: the importance of quality indicators. Curr Med Res Opin.

[3] Gan TJ (2017). Poorly controlled postoperative pain: prevalence, consequences, and prevention. J Pain Res.

[4] Chapman CR, Vierck CJ (2017). The transition of acute postoperative pain to chronic pain: an integrative overview of research on mechanisms. J Pain.

[5] Pasero C, McCaffery M (2007). Orthopaedic postoperative pain management. J Perianesth Nurs.

[6] Harvin J, Kao L (2020). Pain management in the surgical ICU patient. Curr Opin Crit Care.

[7] Garcia JBS, Neto JOB (2020). Living without the opioid epidemic: how far have we come?. Lancet Neurol.

[8] Baker DW (2017). History of the joint commission’s pain standards: lessons for today’s prescription opioid epidemic. JAMA.

[9] Hayes K, Gordon DB (2015). Delivering quality pain management: the challenge for nurses. AORN J.

[10] Ayanian JZ, Markel H (2016). Donabedian’s lasting framework for health care quality. N Engl J Med.

[11] Shoqirat N, Mahasneh D, Dardas L, Singh C, Khresheh R (2019). Nursing documentation of postoperative pain management: a documentary analysis. J Nurs Care Qual.

[12] Peters M, Godfrey C, McInerney P, Munn Z, Tricco A, Khalil H, Aromataris E, Munn Z (2020). JBI Manual for Evidence Synthesis.

[13] Page MJ, McKenzie JE, Bossuyt PM, Boutron I, Hoffmann TC, Mulrow CD (2021). The PRISMA 2020 statement: an updated guideline for reporting systematic reviews. BMJ.

[14] Fontes BV, Moraes ÉB, Hipolito RL, Oliveira AM (2022). Nursing care quality practices in pain management regarding orthopedic surgical patients: a scoping review.

[15] Stavropoulou A, Rovithis M, Kelesi M, Vasilopoulos G, Sigala E, Papageorgiou D (2022). What quality of care means? Exploring clinical nurses’ perceptions on the concept of quality care: a qualitative study. Clin Pract.

[16] Fishman SM, Young HM, Lucas Arwood E, Chou R, Herr K, Murinson BB (2013). Core competencies for pain management: results of an interprofessional consensus summit. Pain Med.

[17] Ouzzani M, Hammady H, Fedorowicz Z, Elmagarmid A (2016). Rayyan: a web and mobile app for systematic reviews. Syst Rev.

[18] Roberts C, Foster D, Shi GG, Lesser E, Heckman M, Whalen J (2019). A collaborative approach to pain control reduces in-hospital opioid use and improves range of motion following total knee Arthroplasty. Cureus.

[19] Smith-Miller CA, Harlos L, Roszell SS, Bechtel GA (2009). A comparison of patient pain responses and medication regimens after hip/knee replacement. Orthop Nurs.

[20] Bell LM (1999). A descriptive study of a perioperative pain service program.. United States Air Force Nurse Corps: Uniformed Services University of the Health Sciences.

[21] Briggs M, Closs JS (1999). A descriptive study of the use of visual analogue scales and verbal rating scales for the assessment of postoperative pain in orthopedic patients. J Pain Symptom Manage.

[22] Ettrich U, Seifert J, Scharnagel R, Günther KP (2007). A multimodal and multidisciplinary postoperative pain management concept. Orthopade.

[23] Pizzi LJ, Bates M, Chelly JE, Goodrich CJ (2020). A prospective randomized trial of an oral patient-controlled analgesia device versus usual care following total hip arthroplasty. Orthop Nurs.

[24] Wilson RA, Watt-Watson J, Hodnett E, Tranmer J (2016). A randomized controlled trial of an individualized preoperative education intervention for symptom management after total knee arthroplasty. Orthop Nurs.

[25] Laframboise-Otto JM, Horodyski M, Parvataneni HK, Horgas AL (2021). A randomized controlled trial of music for pain relief after arthroplasty surgery. Pain Manag Nurs.

[26] Bell F (2000). A review of the literature on the attitudes of nurses to acute pain management. J Orthop Nurs.

[27] Cho Y-H, Kim C-K, Heo K-H, Lee MS, Ha I-H, Son DW (2015). Acupuncture for acute postoperative pain after back surgery: a systematic review and meta-analysis of randomized controlled trials. Pain Pract.

[28] Shin H-R, Park K, Seo J, An S-H, Yeom S-R, Kwon Y-D (2019). Acupuncture for perioperative care of total hip arthroplasty. Medicine (Baltimore).

[29] Moizo E, Berti M, Marchetti C, Deni F, Albertin A, Muzzolon F (2004). Acute Pain Service and multimodal therapy for postsurgical pain control: evaluation of protocol efficacy. Minerva Anestesiol.

[30] Titler MG, Herr K, Schilling ML, Marsh JL, Xie X, Ardery G (2003). Acute pain treatment for older adults hospitalized with hip fracture: current nursing practices and perceived barriers. Appl Nurs Res.

[31] Lin PC (2012). An evaluation of the effectiveness of relaxation therapy for patients receiving joint replacement surgery. J Clin Nurs.

[32] Yu G, Ma S, Zhang X, Liu S, Zhang L, Xu L (2021). Analysis of effect of high-quality nursing on pain of emergency orthopedic trauma patients and related factors affecting postoperative pain. Am J Transl Res.

[33] Xie G, Liu F, Fan L, Wen Y (2022). Analysis of fast-track surgery with pain care on postoperative pain improvement and complication prevention in perioperative spine surgery patients. Emerg Med Int.

[34] Adeboye A, Hart R, Senapathi SH, Ali N, Holman L, Thomas HW (2021). Assessment of functional pain score by comparing to traditional pain scores. Cureus.

[35] Milutinović D, Milovanović V, Pjević M, Martinov-Cvejin M, Cigić T (2009). Assessment of quality of care in acute postoperative pain management. Vojnosanit Pregl.

[36] Paula GR, Reis VS, Ribeiro FA, Gagliazzi MT (2011). Assistência de enfermagem e dor em pacientes ortopédicos na recuperação anestésica, no Brasil. Rev Dor.

[37] Barbosa MH (2014). Araujo NF, Silva JAJ, Corrêa TB, Moreira TM, Andrade ÉV. Avaliação da intensidade da dor e analgesia em pacientes no período pós-operatório de cirurgias ortopédicas. Esc Anna Nery.

[38] Wang T, Chang C, Lou M, Ao M, Liu C, Liang S (2015). Biofeedback relaxation for pain associated with continuous passive motion in Taiwanese patients after total knee arthroplasty: biofeedback relaxation for acute postoperative pain. Res Nurs Health.

[39] Wong EM-L, Chair S-Y, Leung DY, Chan SW-C (2014). Can a brief educational intervention improve sleep and anxiety outcomes for emergency orthopaedic surgical patients?. Contemp Nurse.

[40] Petrie K, Matzkin E (2019). Can pharmacological and non-pharmacological sleep aids reduce post-operative pain and opioid usage? A review of the literature. Orthop Rev (Pavia).

[41] Harper CM, Dong Y, Thornhill TS, Wright J, Ready J, Brick GW (2015). Can therapy dogs improve pain and satisfaction after total joint arthroplasty? A randomized controlled trial. Clin Orthop Relat Res.

[42] Huang N, Cunningham F, Laurito CE, Chen C (2001). Can we do better with postoperative pain management?. Am J Surg.

[43] Myoji Y, Fujita K, Mawatari M, Tabuchi Y (2015). Changes in sleep-wake rhythms, subjective sleep quality and pain among patients undergoing total hip arthroplasty: postoperative sleep and pain. Int J Nurs Pract.

[44] Wang Z-Q, Zhan S-Y, Fransen M, Lin J-H (2012). Clinical attitudes towards pain treatment post-orthopedic surgery: a multicenter study in Beijing. Chin Med J (Engl).

[45] Abou-Setta AM, Beaupre LA, Rashiq S, Dryden DM, Hamm MP, Sadowski CA (2011). Comparative effectiveness of pain management interventions for hip fracture: a systematic review. Ann Intern Med.

[46] DeWaters T, Faut-Callahan M, McCann JJ, Paice JA, Fogg L, Hollinger-Smith L (2008). Comparison of self-reported pain and the PAINAD scale in hospitalized cognitively impaired and intact older adults after hip fracture surgery. Orthop Nurs.

[47] Wainwright TW, Gill M, McDonald DA, Middleton RG, Reed M, Sahota O (2020). Consensus statement for perioperative care in total hip replacement and total knee replacement surgery: Enhanced Recovery After Surgery (ERAS(®)) Society recommendations. Acta Orthop.

[48] Adie S, Kwan A, Naylor JM, Harris IA, Mittal R (2012). Cryotherapy following total knee replacement. Cochrane Database Syst Rev.

[49] Botti M, Kent B, Bucknall T, Duke M, Johnstone M-J, Considine J (2014). Development of a Management Algorithm for Post-operative Pain (MAPP) after total knee and total hip replacement: study rationale and design. Implement Sci.

[50] Weekes DG, Wicks ED, Campbell RE, Hadley C, Carter A, Chaudhry Z (2021). Do relaxation exercises decrease postoperative pain after rotator cuff repair? A randomized controlled trial. Clin Orthop Relat Res.

[51] Wittig-Wells D, Johnson I, Samms-McPherson J, Thankachan S, Titus B, Jacob A (2015). Does the use of a brief cryotherapy intervention with analgesic administration improve pain management after total knee arthroplasty?. Orthop Nurs.

[52] Xue Y, Sun Z, Hu P, Wang F, Zuo L, Chen Y (2018). Effect of a comprehensive rehabilitation nursing program on patients undergoing elbow arthrolysis. Int J Clin Exp Med.

[53] Patiyal N, Kalyani V, Mishra R, Kataria N, Sharma S, Parashar A (2021). Effect of music therapy on pain, anxiety, and use of opioids among patients underwent orthopedic surgery: a systematic review and meta-analysis. Cureus.

[54] Seers K, Crichton N, Tutton L, Smith L, Saunders T (2008). Effectiveness of relaxation for postoperative pain and anxiety: randomized controlled trial. J Adv Nurs.

[55] Ko HF, Chen C-H, Dong K-R, Wu H-C (2021). Effects of acupuncture on postoperative pain after total knee replacement: systematic literature review and meta-analysis. Pain Med.

[56] Qi Y, Hao SN, Zhang J, Zhao CB, Lian Y (2017). Effects of comprehensive nursing on the pain and joint functional recovery of patients with hip replacements. Biomed Res.

[57] Rucinski K, Cook JL (2020). Effects of preoperative opioid education on postoperative opioid use and pain management in orthopaedics: A systematic review. J Orthop.

[58] Duncan K, Pozehl B (2000). Effects of performance feedback on patient pain outcomes. Clin Nurs Res.

[59] Lim YC, Yobas P, Chen HC (2014). Efficacy of relaxation intervention on pain, self-efficacy, and stress-related variables in patients following total knee replacement surgery. Pain Manag Nurs.

[60] Schroeder DL, Hoffman LA, Fioravanti M, Medley DP, Zullo TG, Tuite PK (2016). Enhancing nurses’ pain assessment to improve patient satisfaction. Orthop Nurs.

[61] See MTA, Kowitlawakul Y, Tan AJQ, Liaw SY (2018). Expectations and experiences of patients with osteoarthritis undergoing total joint arthroplasty: an integrative review. Int J Nurs Pract.

[62] Yilmazer T (2017). Expectations that patients have of nurses regarding pain management during the post-operative period and interventions of nurses for patients in pain. Journal of Clinical and Analytical Medicine.

[63] Carpenter JJ, Hines SH, Lan VM (2017). Guided imagery for pain management in postoperative orthopedic patients: an integrative literature review. J Holist Nurs.

[64] Wikström L, Eriksson K, Fridlund B, Årestedt K, Broström A (2016). Healthcare professionals’ descriptions of care experiences and actions when assessing postoperative pain - a critical incident technique analysis. Scand J Caring Sci.

[65] Keast M, Hutchinson AF, Khaw D, McDonall J (2022). Impact of pain on postoperative recovery and participation in care following knee arthroplasty surgery: a qualitative descriptive study. Pain Manag Nurs.

[66] Cui C, Wang L-X, Li Q, Zaslansky R, Li L (2018). Implementing a pain management nursing protocol for orthopaedic surgical patients: results from a PAIN OUT project. J Clin Nurs.

[67] Karlsten R, Ström K, Gunningberg L (2005). Improving assessment of postoperative pain in surgical wards by education and training. Qual Saf Health Care.

[68] Neitzel JJ, Miller EH, Shepherd MF, Belgrade M (1999). Improving pain management after total joint replacement surgery. Orthop Nurs.

[69] Doi K, Shimoda R, Gibbons G (2014). Improving pain management in orthopedic surgical patients with opioid tolerance. Nurs Clin North Am.

[70] Huang CH, Yeh ML, Chen FP, Wu D (2022). Low-level laser acupuncture reduces postoperative pain and morphine consumption in older patients with total knee arthroplasty: a randomized placebo-controlled trial. J Integr Med.

[71] Colwell CW (2008). Management of pain after total knee arthroplasty. Semin Arthroplasty.

[72] Schinsky MF, McCune C, Bonomi J (2016). Multifaceted comparison of two cryotherapy devices used after total knee arthroplasty: cryotherapy device comparison. Orthop Nurs.

[73] Wang C, Tian F (2021). Music intervention to orthopedic patients: a possible alternative solution to control pain. Comput Math Methods Med.

[74] Brown FM (2008). Nursing care after a shoulder arthroplasty. Orthop Nurs.

[75] Specht K, Kjaersgaard-Andersen P, Kehlet H, Pedersen BD (2015). Nursing in fast-track total hip and knee arthroplasty: A retrospective study. Int J Orthop Trauma Nurs.

[76] Hardwick ME, Pulido PA, Adelson WS (2012). Nursing intervention using healing touch in bilateral total knee arthroplasty. Orthop Nurs.

[77] Skokowska B, Bączyk G, Zembroń E, Bielawska A, Gacek L (2016). Ocena jakości opieki pielęgniarskiej w zakresie bólu pooperacyjnego dokonywana przez chorych po zabiegach ortopedycznych. Pielęgniarstwo Polskie.

[78] Nussenzveig TC (1999). Pain management after total joint replacement and its impact on patient outcomes. AORN J.

[79] Colquhoun L, Shepherd V, Neil M (2019). Pain management in new amputees: a nursing perspective. Br J Nurs.

[80] Wang Y, Wang F, Lu C (2020). Pain-free nursing care improves therapeutic outcome for patients with acute bone fracture after orthopedics surgery. Asian J Surg.

[81] McDonall J, de Steiger R, Reynolds J, Redley B, Livingston PM, Hutchinson AF (2019). Patient activation intervention to facilitate participation in recovery after total knee replacement (MIME): a cluster randomised cross-over trial. BMJ Qual Saf.

[82] McDonall J, de Steiger R, Reynolds J, Redley B, Livingston P, Botti M (2016). Patient participation in postoperative care activities in patients undergoing total knee replacement surgery: multimedia Intervention for Managing patient Experience (MIME). Study protocol for a cluster randomised crossover trial. BMC Musculoskelet Disord.

[83] Sjöling M, Nordahl G, Olofsson N, Asplund K (2003). The impact of preoperative information on state anxiety, postoperative pain and satisfaction with pain management. Patient Educ Couns.

[84] Niemi-Murola L, Pöyhiä R, Onkinen K, Rhen B, Mäkelä A, Niemi TT (2007). Patient satisfaction with postoperative pain management—effect of preoperative factors. Pain Manag Nurs.

[85] Stomberg MW, Oman U-B (2006). Patients undergoing total hip arthroplasty: a perioperative pain experience. J Clin Nurs.

[86] Joelsson M, Olsson L-E, Jakobsson E (2010). Patients’ experience of pain and pain relief following hip replacement surgery: pain and pain relief following hip replacement surgery. J Clin Nurs.

[87] Zhu N, Xu P, Ma J, Liang Y, Xu X, Li J (2019). Patients, caregivers and nurses’ attitudes toward patients’ participation in knee and hip joint replacement pain management: a Q-methodology study. Contemp Nurse.

[88] Ingadottir B, Blondal K, Jaarsma T, Thylen I (2016). Perceptions about traditional and novel methods to learn about postoperative pain management: a qualitative study. J Adv Nurs.

[89] Yıldırım M, Çizmeciyan ES, Kaya G, Başaran Z, Şahin Karaman F, Dursun S (2015). Perceptions of pain levels among orthopedic surgery patients, their relatives, and nurses. Agri.

[90] Idvall E, Bergqvist A, Silverhjelm J, Unosson M (2008). Perspectives of Swedish patients on postoperative pain management. Nurs Health Sci.

[91] Arkin LC, Lyons MT, McNaughton MA, Quinlan-Colwell A (2022). Position Statement: acute perioperative pain management among patients undergoing orthopedic surgery by the American Society for Pain Management Nursing and the National Association of Orthopaedic Nurses. Pain Manag Nurs.

[92] Notte BB, Fazzini C, Mooney RA (2016). Reiki’s effect on patients with total knee arthroplasty: a pilot study. Nursing.

[93] Wong EM-L, Chan SW-C, Chair S-Y (2010). The effect of educational intervention on pain beliefs and postoperative pain relief among Chinese patients with fractured limbs. J Clin Nurs.

[94] Chen S-R, Chen C-S, Lin P-C (2014). The effect of educational intervention on the pain and rehabilitation performance of patients who undergo a total knee replacement. J Clin Nurs.

[95] Rahmani M, Bahraminejad N, Rezaei M (2020). The effect of family-oriented educational intervention on postoperative pain after orthopedic surgery. Iran J Nurs Midwifery Res.

[96] Pasyar N, Rambod M, Kahkhaee FR (2018). The effect of foot massage on pain intensity and anxiety in patients having undergone a tibial shaft fracture surgery: A randomized clinical trial. J Orthop Trauma.

[97] Schneider MA (2018). The effect of listening to music on postoperative pain in adult orthopedic patients. J Holist Nurs.

[98] McCaffrey R, Locsin R (2006). The effect of music on pain and acute confusion in older adults undergoing hip and knee surgery. Holist Nurs Pract.

[99] Büyükyılmaz F, Aştı T (2013). The effect of relaxation techniques and back massage on pain and anxiety in Turkish total hip or knee arthroplasty patients. Pain Manag Nurs.

[100] Erden S, Demir SG, Kanatlı U, Danacı F, Carboğa B (2017). The effect of standard pain assessment on pain and analgesic consumption amount in patients undergoing arthroscopic shoulder surgery. Appl Nurs Res.

[101] Elmali H, Balci Akpinar R (2017). The effect of watching funny and unfunny videos on post-surgical pain levels. Complement Ther Clin Pract.

[102] Fang L, Hung C-H, Wu S-L, Fang S-H, Stocker J (2012). The effects of cryotherapy in relieving postarthroscopy pain. J Clin Nurs.

[103] Bahçeli A, Karabulut N (2021). The effects of progressive relaxation exercises following lumbar surgery: a randomized controlled trial. Complement Med Res.

[104] Gatewood CT, Tran AA, Dragoo JL (2017). The efficacy of post-operative devices following knee arthroscopic surgery: a systematic review. Knee Surg Sports Traumatol Arthrosc.

[105] Angelini E, Wolf A, Wijk H, Brisby H, Baranto A (2021). The impact of implementing a person-centred pain management intervention on resistance to change and organizational culture. BMC Health Serv Res.

[106] Sjöling M, Nordahl G (1999). Patient satisfaction with postoperative pain management despite experiencing high levels of pain. J Orthop Nurs.

[107] Andersson V, Otterstrom-Rydberg E, Karlsson A-K (2015). The importance of written and verbal information on pain treatment for patients undergoing surgical interventions. Pain Manag Nurs.

[108] Lambert TL, Cata DM (2014). The traditional method of oral as-needed pain medication delivery compared to an oral patient-controlled analgesia device following total knee arthroplasty. Orthop Nurs.

[109] Antall GF, Kresevic D (2004). The use of guided imagery to manage pain in an elderly orthopaedic population. Orthop Nurs.

[110] Pellino TA, Gordon DB, Engelke ZK, Busse KL, Collins MA, Silver CE (2005). Use of nonpharmacologic interventions for pain and anxiety after total hip and total knee arthroplasty. Orthop Nurs.

[111] Cina-Tschumi B (2007). Evidence-based impact of cryotherapy on postoperative pain, swelling, drainage and tolerance after orthopedic surgery. Pflege.

[112] International Association for the Study of Pain (2024). IASP Curriculum Outline on Pain for Nursing [Internet].

[113] Conselho Federal de Enfermagem (COFEN). (2018). Resolução COFEN Nº 581/2018. Atualiza, no âmbito do Sistema Cofen/Conselhos Regionais de Enfermagem, os procedimentos para Registro de Títulos de PósGraduação Lato e Strictu Sensu concedido a Enfermeiros e aprova a lista das especialidades..

[114] Gordon DB (2015). Acute pain assessment tools. Curr Opin Anaesthesiol.

[115] Small C, Laycock H (2020). Acute postoperative pain management. Br J Surg.

[116] Allen-Duck A, Robinson JC, Stewart MW (2017). Healthcare quality: a concept analysis. Nurs Forum.

[117] Santana MJ, Manalili K, Jolley RJ, Zelinsky S, Quan H, Lu M (2018). How to practice person-centred care: a conceptual framework. Health Expect.

[118] Bhatia A, Buvanendran A (2019). Anesthesia and postoperative pain control: multimodal anesthesia protocol. J Spine Surg.

[119] Montgomery R, McNamara SA (2016). Multimodal pain management for enhanced recovery: reinforcing the shift from traditional pathways through nurse-led interventions. AORN J.

[120] Shraim MA, Massé-Alarie H, Hall LM, Hodges PW (2020). Systematic review and synthesis of mechanism-based classification systems for pain experienced in the musculoskeletal system. Clin J Pain.

