# Supplementary material for: Quality of nursing care in pain management in orthopedic surgical patients: a scoping review
Source: Rev Esc Enferm USP. 2024 Dec 6;58:e20240110. doi: 10.1590/1980-220X-REEUSP-2024-0110en (PMC11649067; doi:10.1590/1980-220X-REEUSP-2024-0110en)
Supplement: Translation

# Translation: Qualidade do cuidado de enfermagem na gestão da dor em pacientes cirúrgicos ortopédicos: revisão de escopo*

**DOI:** 10.1590/1980-220X-REEUSP-2024-0110pt

**Authors:** Bárbara Ventura Fontes, Amanda Magalhaes de Oliveira, Érica Brandão de Moraes, Juliane de Macedo Antunes, Marina de Góes Salvetti, Thalita Gomes do Carmo

**Affiliations:** 1 Universidade Federal Fluminense, Escola de Enfermagem Aurora de Afonso Costa, Niterói, RJ, Brasil.; 2 Instituto Nacional de Traumatologia e Ortopedia, Rio de Janeiro, RJ, Brasil.; 3 Centro Brasileiro para o Cuidado à Saúde Informado por Evidências: Centro de Excelência do Instituto Joanna Briggs, São Paulo, SP, Brasil.; 4 Universidade de São Paulo, Escola de Enfermagem, Departamento de Enfermagem Médico-Cirúrgica. São Paulo, SP, Brasil.

**Keywords:** Enfermagem em Ortopedia e Traumatologia, Qualidade da Assistência à Saúde, Segurança do Paciente, Dor Pós-Operatória, Enfermagem Baseada em Evidências

## Abstract

**Objetivo:**

Mapear as evidências sobre as práticas de qualidade do cuidado de enfermagem na gestão da dor em pacientes cirúrgicos ortopédicos.

**Método:**

Revisão de escopo, conforme as recomendações do Manual JBI. As buscas foram realizadas nas bases de dados MEDLINE (PubMed), LILACS (BVS Regional), Scopus, Embase, Web of Science, Cochrane, Cinahl e literatura cinzenta, independentemente do idioma e período. A seleção e a extração foram realizadas por dois revisores independentes, utilizando critérios de inclusão/exclusão, e os dados extraídos foram organizados para refletir os principais temas ou padrões recorrentes relacionados ao objetivo da revisão.

**Resultados:**

Foram incluídos 94 estudos, a maioria dos Estados Unidos, correspondendo a 34% da amostra, e publicados entre 1997 e 2022. Os achados foram categorizados em práticas de qualidade de enfermagem na gestão da dor relacionadas à organização e monitoramento das unidades, e período pré e pós-operatório.

**Conclusão:**

A pesquisa revelou que as práticas de qualidade do cuidado de enfermagem na gestão da dor em pacientes cirúrgicos ortopédicos abrangem uma variedade de abordagens, desde o uso de práticas não farmacológicas e educação ao paciente até o uso de escalas de avaliação da dor, capacitação da equipe, treinamento, até procedimentos farmacológicos inovadores.

## INTRODUÇÃO

A Associação Internacional para o Estudo da Dor (IASP) atualizou o conceito de dor como uma experiência sensorial e emocional desagradável, associada ou semelhante àquela associada, a uma lesão tecidual real ou potencial ^(1)^. Dentro desse novo conceito, aspectos importantes devem ser considerados: a dor é sempre uma experiência pessoal que é influenciada, em graus variáveis, por fatores biológicos, psicológicos e sociais; dor e nocicepção são fenômenos diferentes, ou seja, a dor não pode ser determinada exclusivamente pela atividade dos neurônios sensitivos; por meio das experiências de vida, as pessoas aprendem o conceito de dor; o relato de uma pessoa sobre uma experiência de dor deve ser respeitado. Embora a dor geralmente cumpra um papel adaptativo, ela pode ter efeitos adversos na função e no bem-estar social e psicológico; e a descrição verbal é apenas um dos vários comportamentos para expressar a dor^(1)^.

Além de seu aspecto subjetivo, a dor integra processos fisiológicos subjacentes que envolvem os sistemas nervoso sensorial e autônomo, catecolaminas circulantes e outros hormônios de resposta ao estresse e respostas do sistema imunológico à sinalização autonômica e hormonal. Clinicamente, os estados de dor podem ser agudos ou crônicos. A dor aguda caracteriza-se como um sintoma e tende a se resolver com a resolução da causa da dor. Já a dor crônica caracteriza-se por um processo patológico, com alterações no sistema de modulação da dor, e sensibilização central^(2)^.

A dor pós-operatória é a forma mais comum de dor aguda, afetando 80% dos pacientes submetidos a cirurgia. O tempo de recuperação após o procedimento impacta diretamente a frequência e a intensidade da dor, que tende a ser mais intensa nas primeiras 24 horas de pós-operatório^(2,3)^. Geralmente, é uma consequência previsível e temporária da lesão física gerada pelo procedimento cirúrgico, uma função biológica protetora que auxilia na recuperação, ao limitar movimentos e comportamentos que podem causar danos adicionais ao tecido, além de uma resposta do sistema imunológico e inflamatório para a recuperação do tecido lesado^(2,4)^.

Pacientes submetidos a cirurgia ortopédica podem apresentar dor em sua forma aguda, crônica ou uma combinação das duas, sendo que menos da metade de todos os pacientes cirúrgicos relatam alívio adequado da dor^(5)^. Em cirurgias ortopédicas, o alívio efetivo da dor permite melhor mobilidade, reabilitação e acelera o retorno às atividades de vida diária^(6)^.

Apesar do maior foco na compreensão e tratamento da dor pós-operatória e do desenvolvimento de recomendações baseadas em evidências e melhores práticas na busca de um manejo da dor eficaz, o controle da dor pós-operatória continua abaixo do ideal, independentemente do sistema hospitalar, tipo de cirurgia ou país^(2,7)^. O controle inadequado da dor durante o pós-operatório pode resultar na cronificação da dor, afetando de forma negativa a qualidade de vida dos pacientes e aumentando os custos do cuidado para os serviços de saúde^(2,3,4,8)^. Com o objetivo de melhorar a qualidade no gerenciamento da dor, instituições acreditadoras, como a *Joint Commission*, buscou estabelecer padrões que incluem práticas de qualidade em cuidados para diversas categorias profissionais, incluindo a enfermagem^(8)^.

A busca pela melhoria constante da qualidade nos cuidados em saúde traz mudanças que geram melhores resultados para os pacientes, integrando elementos da prática clínica que podem ser padronizados em um determinado contexto^(2)^.

No contexto hospitalar, o enfermeiro é o profissional de saúde que mais frequentemente é responsável por avaliar a resposta do paciente à terapêutica utilizada para alívio da dor, pois são os que gerenciam a administração da analgesia prescrita e utilização das práticas não farmacológicas^(9)^. Com isso, a equipe de enfermagem tem um papel fundamental no manejo da dor porque são os profissionais de saúde que atuam na primeira linha no atendimento ao paciente. Sendo assim, a avaliação e o cuidado da dor são habilidades essenciais e é fundamental para a qualidade do cuidado ao paciente^(10)^.

O gerenciamento efetivo da dor é o principal objetivo do cuidado pós-operatório e contribui para recuperação do paciente, retorno às atividades, conforto e sensação de bem- estar e satisfação, e redução do tempo e os custos da internação^(11)^. Apesar de ser consensual entre as evidências científicas e os profissionais de saúde a importância de um manejo da dor com qualidade no pós-operatório, o controle álgico após procedimento cirúrgico continua sendo um grande problema durante a hospitalização, sendo a dor relatada de forma moderada a intensa em 51% dos pacientes^(7)^.

Identificar as práticas de qualidade no manejo da dor contribui para busca de um cuidado de alta qualidade, seguro e eficiente, beneficiando tanto os pacientes quanto os profissionais, sendo fundamental para avançar o conhecimento na área e melhorar os cuidados de saúde^(2,10)^.

Alguns estudos primários foram publicados sobre o tema e uma busca preliminar na base Medical Literature Analysis and Retrieval System Online (MEDLINE), COCHRANE DATABASE E JBI Evidence Synthesis foi conduzida, e nenhuma revisão sistemática ou de escopo sobre o tópico foi identificada. O objetivo deste estudo foi mapear as evidências sobre as práticas de qualidade do cuidado de enfermagem na gestão da dor em pacientes cirúrgicos ortopédicos.

## MÉTODO

### Desenho do Estudo

Trata-se de um estudo do tipo revisão de escopo baseado no referencial teórico proposto e desenvolvido pela JBI, específico para esse tipo de pesquisa^(12)^, sendo relatado de acordo com os pressupostos do *Preferred Reporting Items for Systematic reviews and Meta-Analyses extension or Scoping Reviews* (PRISMA-ScR)^(13)^.

A revisão de escopo adequa-se bem a esta pesquisa, que visa uma abordagem ampla da temática, a fim de identificar lacunas na literatura e subsidiar pesquisas futuras. O protocolo desta revisão foi registrado, conforme recomendações do JBI, na plataforma de registro *Open Science Framework* (https://osf.io/tfwuh) com DOI 10.17605/OSF.IO/TFWUH^(14)^.

### Identificação da Pergunta de Revisão

Para construção da pergunta, utilizou-se o acrônimo PCC (*Population, Concept* e *Context*), recomendado para revisões de escopo. Com base nessas definições, foi estabelecida a questão de pesquisa: “Quais práticas de qualidade do cuidado de enfermagem na gestão da dor em pacientes cirúrgicos ortopédicos são recomendadas e realizadas no contexto hospitalar”?, onde População = Pacientes cirúrgicos ortopédicos; Conceito = Práticas de qualidade de enfermagem em gestão da dor; e Contexto = ambiente hospitalar.

### Critérios de Elegibilidade

População: Foram considerados estudos com pacientes adultos cirúrgicos ortopédicos, que estejam no ambiente hospitalar, durante o período pré e/ou pós-operatório, independentemente da natureza ou extensão da cirurgia realizada. Foram excluídos os estudos que não respondiam à questão de pesquisa.

Conceito: Como conceito, foram considerados estudos contendo práticas de qualidade do cuidado de enfermagem na gestão da dor em procedimentos cirúrgicos ortopédicos. A qualidade do cuidado de enfermagem é definida como todas as práticas que envolvem um cuidado holístico ao paciente, que abrange as questões de atendimento às necessidades do paciente, competência e empatia dos enfermeiros, melhores resultados do paciente e satisfação^(15)^. Para a gestão da dor, foram considerados estudos que descrevessem práticas que estão relacionadas às competências essenciais do enfermeiro no manejo da dor aguda e crônica, que envolvem natureza multidimensional da dor, avaliação e medição da dor, manejo da dor e contexto do manejo da dor^(16)^.

Contexto: O contexto da pesquisa desenvolveu-se no ambiente hospitalar, onde se concentram as atividades de cuidados médicos e tratamentos aos pacientes. Este ambiente é crucial para a compreensão do manejo da dor no período operatório até questões relacionadas à gestão hospitalar e de políticas de saúde.

Tipos de estudo: Considerou desenhos de estudos experimentais e quase experimentais, incluindo ensaios clínicos randomizados e não randomizados, estudos do tipo antes e depois, e séries temporais. Estudos observacionais, incluindo estudos de coorte, estudos caso-controle e estudos transversais, série e relatos de casos, além de protocolos e diretrizes de prática clínica. Revisões de literatura, teses e artigos de texto e opinião de especialistas também foram considerados para inclusão nesta revisão de escopo.

As publicações que não atendiam aos objetivos do estudo, que não continham informações pertinentes ao cenário proposto e que não abordavam o conceito e o contexto escolhidos foram excluídas.

### Estratégia de Busca E Fontes de Informação

A busca ocorreu de maio a outubro de 2023, por dois revisores independentes, e uma estratégia de busca em três etapas foi utilizada para esta revisão. Uma pesquisa inicial limitada no MEDLINE (Pubmed) e CINAHL foi realizada, seguida da análise das palavras do texto contidas no título e no resumo, e os termos de índice usados para descrever o artigo, conforme exemplificado no Quadro 1. Uma pesquisa secundária completa foi realizada em todos os bancos de dados incluídos na revisão: MEDLINE (PubMed), LILACS (BVS Regional), Scopus, Embase, Web of Science, Cochrane e Cinahl, usando as palavras chaves e os termos de índice identificados na pesquisa inicial. Para ajudar a identificar quaisquer estudos adicionais, uma pesquisa de literatura terciária foi realizada examinando as listas de referências de toda a literatura que apreciasse os critérios de inclusão desta revisão. A revisão considerou estudos em qualquer idioma e sem limite de data de publicação. A busca por literatura cinzenta incluiu: Sites de organizações de dor, Biblioteca Digital de Teses e Dissertações, Protocolos e Diretrizes Clínicas reconhecidas por órgãos governamentais e *National Institute for Health and Care Excellence* (NICE).

**Quadro 1:** Busca e mapeamento dos termos realizados na base MEDLINE em 22/05/2023 – Niterói, RJ, Brasil, 2023.

Estratégia de busca		Resultado
#1	“Orthopedic Procedures” [Mesh] OR”Orthopedic Procedure” OR “Procedure, Orthopedic” OR “Procedures, Orthopedic” OR “Orthopedic Surgical Procedures” OR “Orthopedic Surgical Procedure” OR “Procedure, Orthopedic Surgical” OR “Procedures, Orthopedic Surgical” OR “Surgical Procedure, Orthopedic” OR “Surgical Procedures, Orthopedic” OR “Orthopedic Surgery” OR “Orthopedic Surgeries” OR “Surgeries, Orthopedic” OR “Surgery, Orthopedic” OR “Orthopedic Rehabilitation Surgery” OR “Orthopedic Rehabilitation Surgeries” OR “Rehabilitation Surgeries, Orthopedic” OR “Rehabilitation Surgery, Orthopedic” OR “Surgeries, Orthopedic Rehabilitation” OR”Surgery, Orthopedic Rehabilitation”	432.000
#2	(“Pain, Postoperative”[Mesh] OR “Post-surgical Pain”[tiab] OR “Post surgical Pain”[tiab] OR”Postsurgical Pain”[tiab] OR “Post operative Pain”[tiab] OR “Post operative Pain”[tiab] OR “Post-operative Pains”[tiab] OR “Postoperative Pain”[tiab] OR “Chronic Postoperative Pain”[tiab] OR “Chronic Post-surgical Pain”[tiab] OR “Chronic Post surgical Pain”[tiab] OR “Chronic Postsurgical Pain”[tiab] OR “Persistent Postsurgical Pain”[tiab] OR “Chronic Post operative Pain”[tiab] OR “Chronic Post operative Pain”[tiab] OR “Acute Postoperative Pain”[tiab] OR”Acute Post-operative Pain”[tiab] OR “Acute Post operative Pain”[tiab])	62,084
#3	“Orthopedic Nursing”[Mesh] OR “Nursing, Orthopedic”	1,157
#4	#1 AND #2 AND #3	24

### Seleção Dos Estudos

Os registros foram importados para o gerenciador de referências EndNote (Clarivate Analytics, PA, EUA) e os estudos duplicados foram removidos. Em seguida, as referências geradas do EndNote foram exportadas para a plataforma on-line para revisões sistemáticas Rayyan QCRI^(17)^. A partir disso, foi feita a seleção dos estudos em duas etapas por dois revisores independentes. Primeiramente, pela análise dos títulos e resumos e, posteriormente, os estudos selecionados que atenderam os critérios de elegibilidade e que tiveram consenso entre os dois revisores foram lidos na íntegra para inclusão ou exclusão. As divergências que surgiram entre os revisores foram resolvidas por meio de discussão, e não houve necessidade de um terceiro revisor.

### Extração Dos Dados E Apresentação Dos Resultados

As informações dos documentos selecionados para análise foram extraídas por dois revisores, de maneira independente, com o uso de planilhas do Microsoft Excel^®^. Os dados extraídos incluíram detalhes específicos sobre a população, conceito, contexto, métodos de estudo e principais conclusões relevantes para o objetivo da revisão. A primeira parte da extração traçou o perfil dos estudos contendo os seguintes tópicos: Autor e ano de publicação; Local do Estudo; Tipo de Estudo; Tamanho da amostra; Tipo de cirurgia ortopédica e Hospital (tipo e setor de internação). Após a extração dos dados, os achados foram consolidados em uma tabela que destacou as principais características dos estudos incluídos na pesquisa, proporcionando uma visão geral de todo o material utilizado e sua relevância para o tema, e foram descritos acerca de: práticas de qualidade de enfermagem na gestão da dor relacionadas à organização das unidades e monitoramento dos pacientes; práticas de qualidade de enfermagem na gestão da dor no período pré e pós-operatório. Sendo assim, os dados extraídos foram organizados para refletir os principais temas ou padrões recorrentes relacionados ao objetivo da revisão.

Os dados estão apresentados em figura e quadros, acompanhados de uma análise narrativa.

## RESULTADOS

A pesquisa na base de dados recuperou 3962 estudos/registros potencialmente relevantes. Foram excluídos 453 documentos duplicados. Foram analisadas 3509 publicações pelo título e resumo, sendo excluídos 3.263 por não atenderem aos critérios de inclusão. Dessa forma, 246 estudos foram avaliados na íntegra quanto à elegibilidade. Ao final, foram incluídos 94 estudos.^(18 17,18,19,20,21,22,23,24,25,26,27,28,29,30,31,32,33,34,35,36,37,38,39,40,41,42,43,44,45,46,47,48,49,50,51,52,53,54,55,56,57,58,59,60,61,62,63,64,65,66,67,68,69,70,71,72,73,74,75,76,77,78,79,80,81,82,83,84,85,86,87,88,89,90,91,92,93,94,95,96,97,98,99,100,101,102,103,104,105,106,107,108,109,110,111)^, para compor a amostra final da revisão (Figura 1).

**Figura 1:** Fluxograma do PRISMA-ScR de seleção das publicações^(13)^ – Niterói, RJ, Brasil, 2023.

### Qaa2’

O Quadro 2 apresenta a síntese dos estudos. Dos 94 estudos incluídos, 90 foram publicados em inglês^(18,19,20,21,23,24,25,26,27,28,29,30,31,32,33,34,35,38,39,40,41,42,43,44,45,46,47,48,49,50,51,52,53,54,55,56,57,58,59,60,61,62,63,64,65,66,67,68,69,70,71,72,73,74,75,76,78,79,80,81,82,83,84,85,86,87,88,89,90,91,92,93,94,95,96,97,98,99,100,101,102,103,104,105,106,107,108,109,110,111)^, dois em português^(36,37)^, um em alemão^(22)^ e um em polonês^(77)^. Quanto ao período de publicação, os estudos encontram-se entre os anos de 1997 e 2022, com maior crescimento nas produções a partir de 2015. Quanto à origem, trinta e seis foram produzidos no continente americano^(18,19,20,21,23,24,25,30,34,36,37,40,41,42,45,46,50,51,58,59,60,61,63,68,69,71,72,74,76,78,91,92,97,98,104,108,109,110)^, vinte e nove no continente asiático^(11,12,15,16,17,22,23,27,28,36,37,39,40,41,50,54,57,64,71,77,78,79,86)^, vinte e quatro no continente europeu^(22,26,29,35,62,64,67,75,77,83,84,85,86,88,89,90,91,99,100,101,103,105,106,107,111)^ e cinco na Oceania^(48,49,66,81,82)^. Os países com maior volume de publicações foram: Estados Unidos da América com trinta e dois (34%), China com quinze (16%) e Suécia com nove (8,5%). Dentre as publicações analisadas, os estudos de revisão destacaram-se como os mais utilizados, compreendendo 35,7% do total, seguidos pelos ensaios clínicos randomizados (25,7%). Na pesquisa em outras fontes de dados o que foi recuperado foi excluído por não atender aos critérios de inclusão; na busca em base de dados, foram incluídas duas recomendações^(78,91)^, um consenso^(47)^ e uma dissertação de mestrado^(20)^.

**Quadro 2:** Perfil dos estudos selecionados – Niterói, RJ, Brasil, 2023.

Artigo	Autor/ano/país	Tipo de cirurgia	Tipo de Estudo	Amostra	Local
A1^(18)^	Roberts et al., 2019 Estados Unidos	Artroplastia de joelho	Revisão de Literatura	n = 236	Internação, cirurgia eletiva
A2^(19)^	Smith-Miller et al., 2009 Estados Unidos	Cirurgia Eletiva de quadril/joelho pela primeira vez	Revisões retrospectivas de gráficos	n = 65	Internação, cirurgia eletiva
A3^(20)^	Bell, 1999 Estados Unidos	Quadril e joelho	Dissertação de mestrado	n = 133	Internação, cirurgia eletiva
A4^(21)^	Briggs e Closs, 1999 Estados Unidos	Diversos tipos de cirurgias ortopédicas	Transversal descritivo	n = 417	Enfermaria ortopédica, trauma e cirurgia eletiva
A5^(22)^	Ettrich et al., 2007 Alemanha	Diversos tipos de cirurgias ortopédicas	Revisão de Literatura	Não relata	Emergência e cirurgia eletiva
A6^(23)^	Pizzi et al., 2020 Estados Unidos	Artroplastia de quadril	Ensaio Clínico Randomizado	n = 30	Hospital universitário, unidade ortopédica
A7^(24)^	Wilson et al., 2016 Canadá	Artroplastia de joelho	Ensaio Clínico Randomizado	n = 143	Internação, cirurgia eletiva
A8^(25)^	Laframboise-Otto et al., 2021 Estados Unidos	Artroplastia de joelho e quadril	Ensaio Clínico Randomizado	n = 50	Hospital universitário, unidade ortopédica
A9^(26)^	Bell, 2000 Irlanda	Diversos tipos de cirurgias ortopédicas	Revisão de literatura	n = 56	Não se aplica
A10^(27)^	Cho et al., 2015 Coreia do Sul	Cirurgia de coluna	Revisão sistemática	n = 5	Internação, cirurgia eletiva
A11^(28)^	Shin et al., 2019 Coreia do Sul	Artroplastia de quadril	Revisão Sistemática	n = 24	Não se aplica
A12^(29)^	Moizo et al., 2003 Itália	Diversos tipos de cirurgias ortopédicas	Observacional prospectivo	n = 592	Hospital de cuidados cirúrgicos
A13^(30)^	Titler et al., 2003 Estados Unidos	Artroplastia de quadril	Experimental	n = 709	Hospital de Cuidados Intensivos
A14^(31)^	Lin, 2012 Taiwan	Artroplastia de joelho e quadril	Estudo de Caso controle	n = 93	Centro Médico
A15^(32)^	Yu et al., 2021 China	Trauma	Estudo de Caso-controle	n = 78	Emergência de trauma ortopédico
A16^(33)^	Xie et al., 2022 China	Coluna	Ensaio Clínico Randomizado	n = 126	Hospital geral; cirurgia eletiva
A17^(34)^	Adeboye et al., 2021 Estados Unidos	Artroplastia de joelho /Artrodese coluna	Estudo de coorte	n = 49	Emergência
A18^(35)^	Milutinović et al., 2009 Sérvia	Orto Plástica genital; Orto Plástica coxa, Ligamentoplastia	Estudo Transversal	n = 135	Instituto de Saúde Pública
A19^(36)^	Paula et al., 2011 Brasil	Quadril, fêmur, joelho	Revisão de literatura	n = 18	Hospital Geral e SRPA
A20^(37)^	Barbosa, 2014 Brasil	Cirurgias eletivas, exceto cabeça, pescoço, neurológicas e submetidos à anestesia local.	Estudo prospectivo	n = 132	Hospital Universitário
A21^(38)^	Wang et al., 2015 China	Artroplastia de joelho	Desenho quase experimental	n = 66	Hospital Geral
A22^(39)^	Wong et al., 2014 China	Trauma: quadril ou fêmur	Desenho quase experimental	n = 150	Hospital Geral; Enfermaria
A23^(40)^	Petrie e Matzkin, 2019 Estados Unidos	Artroplastia de joelho e quadril	Revisão de Literatura	n = 10	Hospitalar
A24^(41)^	Harper et al., 2015 Estados Unidos	Artroplastia de joelho e quadril	Ensaio Clínico Randomizado	n = 72	Hospital terceirizado
A25^(42)^	Huang et al., 2001 Estados Unidos	Diversos tipos de cirurgias ortopédicas	Revisão de Literatura	n = 39	Hospital geral; Enfermaria e Ambulatório
A26^(43)^	Myoji et al., 2015 Japão	Artroplastia de quadril	Estudo de Caso-controle	n = 34	Hospital Geral
A27^(44)^	Wang et al., 2012 China	Diversos tipos de cirurgias ortopédicas	Estudo Transversal	n = 1131	Hospital geral; Enfermaria Nível II e III
A28^(45)^	Abou-Setta et al., 2011 Canadá	Fratura de quadril	Revisão Sistemática	83 estudos	Hospital geral; Enfermaria
A29^(46)^	DeWaters et al., 2008 Estados Unidos	Artroplastia de joelho e quadril	Desenho correlacional descritivo.	n = 25	Hospital geral; Ortopedia
A30^(47)^	Wainwright et al., 2020 Inglaterra	Artroplastia de joelho e quadril	Consenso	17 Tópicos de pesquisa	Hospital geral; Enfermaria
A31^(48)^	Adie et al., 2012 Austrália	Artroplastia de joelho	Revisão Sistemática	12 estudos: n = 809	Hospital geral; Enfermaria
A32^(49)^	Botti et al., 2014 Austrália	Artroplastia de joelho e quadril	Estudo multicêntrico	n = 374	Hospital geral; Enfermaria
A33^(50)^	Weekes et al., 2019 Estados Unidos	Reparação artroscópica do manguito rotador	Ensaio clínico randomizado	n = 151	Hospital geral; Enfermaria
A34^(51)^	Wittig-Wells et al., 2015 Estados Unidos	Artroplastia de joelho	Ensaio clínico randomizado	n = 29	Hospital Ortopédico
A35^(52)^	Xue et al., 2018 China	Artroplastia unilateral de cotovelo	Ensaio clínico randomizado	n = 60	Hospital universitário; Enfermaria
A36^(53)^	Patiyal et al., 2021 Indonésia	Artroplastia de joelho	Revisão Sistemática	n = 778	Hospital Geral; Enfermaria
A37^(54)^	Seers et al., 2008 Inglaterra	Artroplastia de joelho e quadril	Ensaio Clínico Randomizado	n = 118	Hospital Ortopédico; Enfermaria
A38^(55)^	Ko et al., 2021 China	Artroplastia de joelho	Revisão Sistemática	n = 891	Hospital Geral; Enfermaria
A39^(56)^	Qi et al., 2017 China	Artroplastia de quadril	Estudo caso-controle	n = 100	Departamento cirúrgico
A40^(57)^	Rucinski K, 2020 Estados Unidos	Artroplastia de joelho e quadril	Revisão Sistemática	n = 778	Hospital Geral; Enfermaria
A41^(58)^	Duncan e Pozehl, 2000 India	Artroplastia de joelho	Quase experimental	n = 240	Hospital Geral; Enfermaria
A42^(59)^	Lim et al., 2014 Singapura	Artroplastia de joelho	Quase experimental	n = 18	Hospital Geral; Enfermaria
A43^(60)^	Schroeder et al., 2016 Estados Unidos	Artroplastia de joelho	Design único, pré/pós-intervenção	n = 30 enfermeiros n = 100 pacientes	Hospital Geral; Unidade de Ortopedia
A44^(61)^	See et al., 2017 Singapura	Artroplastia de joelho e quadril	Revisão sistemática	20 artigos	Hospital Geral; Enfermaria
A45^(62)^	Yılmazer, 2017 Turquia	Diversos tipos de cirurgias ortopédicas	Descritivo	n = 63 enfermeiros n = 315 pacientes	Clínica Ortopédica; Internação
A46^(63)^	Carpenter et al., 2017 Estados Unidos	Diversos tipos de cirurgias ortopédicas	Revisão Integrativa	9 artigos	Hospital Geral; Enfermaria
A47^(64)^	Wikström et al., 2016 Suécia	Diversos tipos de cirurgias ortopédicas	Design exploratório	n = 24	Hospital Geral e Unidade de Ortopedia
A48^(65)^	Keast et al., 2022 Austrália	Artroplastia de joelho	Qualitativo exploratório-descritivo	n = 120	Hospital Geral; Enfermaria
A49^(66)^	Cui et al., 2018 China	Cirurgia ortopédica articular eletiva,	Amostra intervencionista separada, pré e pós-teste	n = 77	Hospital Geral; Enfermaria
A50^(67)^	Karlsten et al., 2005 Suécia	Diversos tipos de cirurgias ortopédicas	Estudo qualitativo descritivo	n = 270	Hospital Universitário; cirurgia geral e ortopedia
A51^(68)^	Neitzel et al., 1999 Estados Unidos	Artroplastia de joelho e quadril	Revisão de Literatura - Prática Baseada em Evidências	n = 118	Hospital Geral; Enfermaria
A52^(69)^	Doi et al., 2014 Estados Unidos	Artroplastia de joelho e quadril	Revisão de Literatura - Prática Baseada em Evidências	n = 63	Hospital Geral; Enfermaria
A53^(70)^	Huang et al., 2022 China	Artroplastia de joelho	Ensaio Clínico Randomizado	n = 82	Hospital Geral; Enfermaria
A54^(71)^	Colwell Jr, 2008 Estados Unidos	Artroplastia de joelho	Revisão de Literatura	n = 50	Hospital Geral; Enfermaria
A55^(72)^	Schinsky et al., 2016 Estados Unidos	Artroplastia de joelho	Estudo prospectivo	n = 100	Hospital Geral; Enfermaria
A56^(73)^	Wang e Tian, 2021 China	Cirurgia de substituição do quadril	Quase experimental	n = 38	Hospital de Ortopedia; Enfermaria e centro de trauma
A57^(74)^	Brown Jr, 2008 Estados Unidos	Artroplastias de ombro	Revisão de literatura	n = 24	Não se aplica
A58^(75)^	Specht et al., 2015 Dinamarca	Artroplastia de joelho e quadril	Desenho retrospectivo e descritivo	n = 310	Hospital Geral
A59^(76)^	Hardwick et al., 2012 Estados Unidos	Artroplastia de joelho	Ensaio Clínico Randomizado	n = 41	Hospital Geral
A60^(77)^	Skokowska et al., 2016 Polônia	Artroplastia de quadril e artroscopia	Revisão da literatura prática baseada em evidências.	n = 112	Pronto-socorro e ortopedia
A61^(78)^	Nussenzveig, 1999 Estados Unidos	Artroplastia de joelho e quadril	Relato de experiência	20 prontuários	Enfermarias pós-operatório
A62^(79)^	Colquhoun et al., 2019 Escócia	Amputação	Implementação	–	Profissionais ligados ao NHS Tayside Pain Service
A63^(80)^	Wang et al., 2020 China	Tratamento de fratura após trauma	Caso-controle	n = 220	Pós-operatório de trauma
A64^(81)^	McDonall et al., 2019 Austrália	Artroplastia de joelho	Randomizado por cluster, cruzado de quatro períodos e avaliação de processo simultâneo	n = 241	Hospital Geral; Enfermaria
A65^(82)^	McDonall et al., 2016 Austrália	Artroplastia de joelho	Ensaio cruzado, processo simultâneo e randomizado.	n = 240	Enfermarias ortopédicas
A66^(83)^	Sjöling et al., 1997 Suécia	Artroplastia de joelho e quadril	Implementação	n = 59	Enfermarias pós-operatório
A67^(84)^	Niemi-Murola et al., 2007 Finlandia	Artroplastia de joelho e quadril	Descritivo	n = 77 pacientes n = 63 enfermeiros	Enfermarias pós-operatório
A68^(85)^	Stomberg e Oman, 2006 Suécia	Artroplastia de quadril	Descritivo e comparativo	n = 112	Enfermaria pós-operatório
A69^(86)^	Joelsson et al., 2009 Suécia	Artroplastia de quadril	Descritivo	n = 50	Enfermaria ortopédica
A70^(87)^	Zhu et al., 2019 China	Artroplastia de joelho e quadril	Qualitativo	n = 45	Enfermaria pós op cirurgia ortopédica
A71^(88)^	Ingadottir et al., 2016 Islândia	Não especificou	Qualitativo descritivo	n = 13	Hospital universitário
A72^(89)^	Yıldırım et al., 2015 Turquia	Não especificou	Estudo quantitativo, descritivo e comparativo	n = 350	Clínicas de Ortopedia
A73^(90)^	Idvall et al., 2008 Suécia	Não especificou	A abordagem qualitativa e descritiva usando entrevistas individuais dos pacientes	n = 30	Enfermarias cirurgia ortopédica e geral
A74^(91)^	Arkin et al., 2022 Estados Unidos	Cirurgia ortopédica	Recomendação de sociedade de especialistas	n = 30	Cuidados pós-operatórios
A75^(92)^	Notte et al., 2016 Estados Unidos	Artroplastia de joelho	Estudo piloto	n = 43	Cuidados pós-operatórios
A76^(93)^	Wong et al., 2010 China	Trauma - Fratura de membros	Quase experimental.	n = 125	Hospital Geral; Enfermaria
A77^(94)^	Chen et al., 2013 China	Artroplastia de joelho	Quase experimental.	n = 92	Enfermaria de ortopedia de um hospital
A78^(95)^	Rahmani et al., 2019 Iran	Artroplastia de joelho	Ensaio clínico não randomizado com grupo controle	n = 46	Enfermaria de ortopedia de um hospital
A79^(96)^	Pasyar et al., 2018 Iran	Trauma - Fratura de membros	Ensaio clínico randomizado	n = 66	Enfermaria de ortopedia de um hospital
A80^(97)^	Schneider, 2016 Estados Unidos	Artroplastia de joelho	Descritivo, comparativo e quase experimental.	n = 65	Unidade hospitalar de agudos que inclui uma população de pacientes ortopédicos
A81^(98)^	McCaffrey e Locsin 2006 Estados Unidos	Cirurgias de quadril /Joelho	Ensaio clínico randomizado e controlado.	Grupo experimental n = 62 Grupo controle n = 62	Enfermaria de ortopedia
A82^(99)^	Büyükyılmaz e Aştı, 2013 Turquia	Artroplastia de joelho e quadril	Desenho experimental	n = 60	Hospital Universitário, enfermaria de ortopedia e trauma
A83^(100)^	Erden, et al., 2016 Turquia	Cirurgia artroscópica do ombro.	Desenho experimental	n = 101	Hospital dia
A84^(101)^	Elmali e Balci Akpinar, 2016 Turquia	Não especificou	Desenho experimental	n = 90	Enfermaria de ortopedia e trauma
A85^(102)^	Fang et al., 2010 Taiwan	Artroscopia	Estudo prospectivo, duplo-cego, quase experimental.	n = 59	Hospital Geral
A86^(103)^	Bahçelia e Karabulut, 2020 Turquia	Microdiscectomia lombar	Estudo experimental com grupo controle e desenho de ensaio clínico randomizado.	n = 97	Enfermaria de pós-operatório
A87^(104)^	Gatewood et al., 2016 Estados Unidos	Artroplastia de joelho	Revisão sistemática	n = 37	Cuidados pós-operatórios
A88^(105)^	Angelini et al., 2021 Suécia	Cirurgia eletiva da coluna lombar	Implementação	n = 81	Enfermaria
A89^(106)^	Sjo ¨linga e Nordahl, 2002 Suécia	Artroplastia de joelho	Desenho experimental prospectivo com dois grupos paralelos	n = 60	Unidade ortopédica
A90^(107)^	Andersson et al., 2015 Suécia	Artroplastia de joelho e quadril	Estudo qualitativo descritivo	n = 18	Unidade ortopédica
A91^(108)^	Lambert e Cata, 2014 Estados Unidos	Artroplastia de joelho e quadril	Estudo comparativo quantitativo.	n = 30	Unidade ortopédica
A92^(109)^	Antall e Kresevic, 2004 Estados Unidos	Artroplastia de joelho e quadril	Desenho experimental	n = 13	Unidade ortopédica
A93^(110)^	Pellino et al., 2005 Estados Unidos	Artroplastia de joelho e quadril	Desenho descritivo comparativo e correlacional.	65 prontuários	Unidade ortopédica
A94^(111)^	Cina-Tschumi, 2007 Alemanha	Joelho/Quadril/Coluna/Ombro	Revisão de literatura	17 artigos	Hospital Geral; Enfermaria

As práticas de qualidade na gestão da dor encontradas nos estudos foram classificadas em organização das unidades e monitoramento do paciente e quanto ao período operatório em que foram aplicadas (Quadro 3). Com relação à organização das unidades e monitoramento do paciente, as pesquisas utilizaram como intervenção a capacitação da equipe de enfermagem, a prática baseada em evidências, o uso de protocolos e planejamento da assistência; foram realizadas independente do tempo operatório e tinham como objetivo o manejo da dor a partir da organização dos serviços.

**Quadro 3:** Práticas de qualidade de enfermagem na gestão da dor - Niterói, RJ, Brasil, ٢٠٢٣.

Práticas de Qualidade de enfermagem na gestão da dor	
**Organização das unidades e monitoramento do paciente**	Educação para o autogerenciamento da dor^(24,25)^; Uso de escalas de avaliação da dor ^(30)^; Cuidado individualizado: Gerenciar qualidade do sono: ^(26,29)^; Participação do paciente nas decisões do cuidado^(23, 55)^; Procedimentos farmacológicos/novos: Analgesia controlada pelo paciente^(23)^; Equipe especializada: Enfermagem especializada na dor^(56,82,89,103,105,109)^; Equipe multidisciplinar^(35,44,59,62,105)^; Enfermagem de qualidade ^(32,74,75)^; Gerenciamento: Capacitação da equipe^(36,37,47,52, 55,56,57,60,62,67,69,70,73,84,87,88, 89,91,92,103,109)^; Protocolo padrão e educação ^(19,22,26,29,36,38,41,42,44,45,63,65, 97, 100,107)^; Prática Baseada em Evidências^(29,48,49,50,65,66,67,75,80,87,103,106,110)^; Planejamento de Assistência^(22,26,32,85,89,104)^; Uso de algoritmo^(34,48)^; Avaliação por meio de auditorias ^(64,89)^; Feedback de desempenho individual^(58)^; Condições de trabalho na avaliação da dor:^(77)^; Fluxograma^(79)^; Uso de checklist^(105)^; Fornecimento de kit de tratamento não farmacológico^(110)^; Criação de guia específico para crioterapia^(111)^;
**Período pré-operatório**	Uso de práticas não farmacológicas para alívio da dor: Acupuntura^(60)^; Musicoterapia^(25,53)^; Terapia relaxante (imagens guiadas e meditação)^(31,54,63)^; Respiração profunda^(39,60)^; Crioterapia^(44)^; Neuroestimulação elétrica transcutânea^(45)^; Terapia Reiki^(92)^; Educação para o autogerenciamento da dor^(22,24,32,33,39,42,44,47,50,56, 57,61,71,83,85, 87,89,91,93,94,95,106)^; Uso de escalas de avaliação da dor^(18,21,37,43,95,56,69,75,85)^; Cuidado individualizado: Gerenciar qualidade do sono^(43)^; Participação do paciente nas decisões do cuidado^(33,57,68)^; Procedimentos farmacológicos/novos: Terapia multimodal medicamentosa^(56)^; Gerenciamento: Assistência^(37)^;
**Período pós-operatório**	Uso de práticas não farmacológicas para alívio da dor: Crioterapia^(48,50,51,52,60,62,69,72,102,104,110,111)^; Musicoterapia^(25,36,53,60,73,97,98,99)^; Terapia relaxante (Imagens guiadas e meditação^(22,31,36,38,54,63,69,109)^; Acupuntura^(22,24,27,28,56)^; Massagem^(24, 62, 96, 97,110)^; Reposicionamento e deambulação precoce^(33,62,69, 100)^; Respiração profunda^(39,99)^; Hipertermoterapia^(46, 83)^; Terapia vibracional^(36)^; Aromaterapia^(36)^; Cãoterapia ^(41)^; Neuroestimulação elétrica transcutânea^(45)^; Fortalecimento espiritual^(60)^; Acupuntura a laser de baixa intensidade^(70)^; Toque de Cura (HT) ^(76)^; Terapia Reiki^(92)^; Técnica cognitivo-comportamental de distração (Assistir a vídeos engraçados)^(101)^; Educação para o autogerenciamento da dor:^(20, 44,57,59,61,68,74,75,82, 87, 106)^; Uso de cartilha^(95,107)^ Abordagem cognitivo-comportamental^(93)^; Uso de escalas de avaliação da dor^(18,21,23,34,42,43,44,46,49,56, 64,66,68,78,79, 81,91,95,100)^;
	Participação do paciente nas decisões do cuidado^(56,57,65,81,82,85,91,106)^; Procedimentos farmacológicos/novos: Terapia multimodal medicamentosa^(19,29,37, 45,47,69,71)^; Analgesia controlada pelo paciente^(67,71,74,75,78,108)^; Pré-analgesia na unidade de recuperação pós-anestésica^(23)^; Gerenciamento: Feedback de desempenho individual^(58)^;

Quanto ao período em que as melhores práticas foram utilizadas, observamos que as intervenções estudadas por vezes se repetem, como o uso de algumas práticas não farmacológicas; sendo assim, outra classificação realizada foi em relação ao período operatório (pré e pós-operatório).

A extração de dados revelou uma série de práticas relevantes associadas à gestão da dor, dentre elas, o destaque foi a utilização de abordagens não farmacológicas para o alívio da dor, evidenciada em 34,4% dos casos. Além disso, foram identificados casos de gerenciamento eficaz (14,6%), educação para o autogerenciamento da dor (13,5%), adoção de procedimentos inovadores (10,4%), formação de equipes especializadas no gerenciamento da dor (10,4%), uso de escalas de avaliação da dor (5,2%), atenção individualizada (4,2%), e participação ativa do paciente nas decisões do cuidado (7,3%).

## DISCUSSÃO

Nesta revisão, foi possível mapear as práticas de qualidade do cuidado de enfermagem na gestão da dor em pacientes cirúrgicos ortopédicos. A qualidade do cuidado é um elemento essencial para a assistência à saúde, e os enfermeiros desempenham um papel vital na gestão do cuidado na dor pós-operatória, garantindo que os pacientes recebam alívio adequado e individualizado da dor, promovendo seu bem-estar geral e recuperação.

A IASP também define um currículo mínimo que reúne conhecimentos que o enfermeiro deve adquirir para que haja uma adequada gestão da dor^(112)^. No Brasil, a atuação do enfermeiro no manejo da dor está regulamentada pela Resolução Cofen 581/2018^(113)^.

Os estudos mencionam a importância de uma equipe de enfermagem especializada em dor^(56,82,89,103,105,109)^, bem como a importância da atuação de equipes multidisciplinares^(35,44,59,62,105)^. Sendo o controle da dor um importante resultado a ser acompanhado quando pensamos na qualidade e segurança da assistência, e que, apesar disso, pacientes internados ainda tem a sua dor tratada de forma ineficaz, pesquisas que tenham como foco estratégias que possam impactar o manejo da dor realizado são essenciais para transformar a prática^(9,113,114)^.

Uma quantidade considerável dos estudos selecionados estava voltada para a capacitação profissional, prática baseada em evidências, definição de protocolos assistenciais e uso de auditorias para avaliar a prática utilizada como forma de intervenção para tornar o manejo da dor realizado por enfermeiros efetivo, seguro e baseado nas melhores práticas.

A organização das unidades e o monitoramento do paciente após a cirurgia são cruciais para assegurar a eficácia do tratamento e a segurança. Isso inclui várias práticas de gerenciamento encontradas nos estudos, como: capacitação da equipe^(36,37,47,52,55,56,57,60,62,67,69,70,73,84,87,88,89,91,92,103,109)^, implementação de protocolo padrão e educação^(19,22,26,29,36,38,41,42,44,45,63,65,97,100,107)^, prática baseada em evidências^(30,49,50,51,66,67,68,76,80,88,104,107,111)^, planejamento de assistência^(23,27,33,86,90,104)^, uso de algoritmo^(34,48)^, avaliação por meio de auditorias^(64,89)^, condições de trabalho na avaliação da dor^(77)^, fluxograma^(79)^, uso de checklist^(105)^, fornecimento de kit de tratamento não farmacológico^(110)^ e criação de guia específico para crioterapia^(111)^. O objetivo dessas estratégias é incentivar uma abordagem sistemática e eficiente para o tratamento da dor após a cirurgia, assegurando a qualidade do cuidado e a melhoria contínua dos processos clínicos.

É relevante que todas as práticas de qualidade estejam fundamentadas nas evidências científicas, e que um protocolo institucionalizado seja instituído, padronizando condutas e fluxos no atendimento. A avaliação da dor, com uso de instrumentos validados e padronizados na instituição foi citada em diversos estudos^(18,21,23,34,37,42,43,44,46,49,56,63,66,68,69,75,78,79,81,91,95,100)^. A avaliação e prestação de cuidados eficazes e seguros refletem-se em uma cultura de excelência e resultam na obtenção de resultados desejados^(115,116)^. Um dos autores apresentou uma recomendação diferenciada das relatadas, que é o feedback de desempenho individual, um processo em que os profissionais de saúde recebem informações específicas sobre a qualidade e eficácia de suas práticas relacionadas ao controle da dor em pacientes e os resultados indicaram que fornecer feedback aos enfermeiros sobre as suas práticas anteriores de gestão da dor pode melhorar os resultados da dor pós-operatória dos pacientes^(58)^.

Durante o período pré e pós-operatório os estudos mostraram a relevância de um tratamento multimodal da dor, envolvendo abordagem farmacológica e não farmacológica na gestão da dor. Essa abordagem pode minimizar a quantidade administrada de opioides e riscos associados. Apesar disso, muitos pacientes cirúrgicos ainda recebem apenas o tratamento farmacológico para a dor. Dentro desse contexto, o enfermeiro tem papel fundamental na implementação de práticas não farmacológicas para a gestão da dor nos serviços de saúde. No período pré-operatório, diversas práticas não farmacológicas foram empregadas nos estudos, incluindo acupuntura^(23)^, musicoterapia^(25,53,57)^, terapia relaxante (como imagens guiadas e meditação)^(31,54,63)^, respiração profunda^(39,60)^, crioterapia^(45)^, neuroestimulação transcutânea^(45)^ e terapia reiki^(92)^. No período pós-operatório, as mais utilizadas no alívio da dor incluíram crioterapia^(48,50,51,52,60,62,69,72,102,104,110,111)^, musicoterapia^(25,36,53,57,60,73,97,98,99)^, terapia relaxante (como imagens guiadas e meditação)^(22,31,36,38,54,63,69,109)^, acupuntura^(22,24,27,28,55)^, massagem^(24,62,96,99,110)^, reposicionamento e deambulação precoce^(33,62,69,100)^, respiração profunda^(39,99)^, hipertermoterapia^(46,83)^, terapia vibracional^(20)^, aromaterapia^(36)^, cãoterapia^(41)^, neuroestimulação elétrica transcutânea^(45)^, fortalecimento espiritual^(60)^, acupuntura a laser de baixa intensidade^(70)^, toque de cura (HT)^(76)^ e terapia reiki^(92)^.

Os estudos também enfatizaram a importância do cuidado centrado no paciente. Este é um modelo de cuidado que coloca o paciente no centro do processo de tomada de decisão, considerando as suas necessidades, desejos e valores individuais ao planejar a assistência^(117)^. Esse modelo, além de fortalecer a segurança nos processos de cuidado, gera impacto no manejo da dor, tendo em vista a multidimensionalidade e subjetividade da dor. Dessa forma, a educação para o autogerenciamento da dor foi citada em vários estudos destacando a sua importância^(22,24,32,33,39,42,44,47,50,56,62,71,83,85,87,89,91,93,94,95,106)^, inclusive como política educacional da instituição para o autocontrole da dor^(20,44,59,61,68,74,75,82,87,106)^.

Outros estudos trouxeram o cuidado individualizado com ênfase na participação ativa do paciente nas decisões do cuidado^(32,56,65,68,81,82,85,91,106)^ e no gerenciamento da qualidade do sono^(39,40,41,43)^, incluindo o uso de cartilhas^(95,107)^ e a abordagem cognitivo-comportamental^(101)^ como estratégias. Essas práticas visam otimizar o manejo da dor pré-operatória e melhorar a experiência do paciente durante o processo cirúrgico ortopédico.

Alguns estudos comprovaram que o mecanismo da dor não é igual, ocorre de forma diferente para cada pessoa, sendo considerada uma experiência pessoal e intransferível^(118,119,120)^, um fenômeno multidimensional e subjetivo, o que torna a sua avaliação um processo complexo, visto que não pode ser medida de forma objetiva^(120)^. Assim, os estudos selecionados nesta revisão abordam justamente a educação para autogerenciamento da dor, participação do paciente nas decisões do cuidado e cuidado individualizado, capacitação da equipe e avaliação e monitoramento dos pacientes, evidenciando o protagonismo do enfermeiro no manejo da dor, no sentido de que quando realizam uma gestão da dor bem estruturada e individualizada os resultados para o controle da dor e satisfação do paciente são positivos.

Existem novos procedimentos farmacológicos, como terapia multimodal medicamentosa^(19,29,37,45,47,69,71)^, analgesia controlada pelo paciente^(67,71,74,75,78,108)^ e a pré-analgesia na unidade de recuperação pós-anestésica^(23)^. O uso de escalas de avaliação da dor é comum para monitorar e quantificar a dor dos pacientes, proporcionando uma base objetiva para o tratamento^(18,21,37,43,56,69,75,85,95)^. Essas abordagens multidimensionais têm como objetivo aperfeiçoar o manejo da dor pós-operatória e proporcionar uma melhor recuperação para os pacientes.

Como limitação, este estudo, por ser uma revisão de escopo, não avaliou a qualidade e risco de viés dos estudos primários e, portanto, não traz recomendações sobre a efetividade das práticas citadas para a gestão da dor. No entanto, o estudo possibilita um panorama das práticas evidenciadas na literatura, trazendo o enfermeiro como protagonista de práticas de qualidade na gestão da dor em pacientes cirúrgicos ortopédicos. Este estudo também contribui para o fortalecimento das práticas não farmacológicas à medida que traz diversas possibilidades de uso para o paciente cirúrgico.

## CONCLUSÃO

Os resultados desta revisão destacam a complexidade e a importância do manejo da dor em pacientes submetidos à cirurgia ortopédica. A dor pós-operatória foi considerada um desafio significativo que afeta o bem-estar e a recuperação desses pacientes. Ao analisar uma variedade de fontes, incluindo estudos experimentais e observacionais, protocolos, diretrizes clínicas e literatura cinzenta, conseguimos reunir uma ampla gama de informações valiosas. As práticas de qualidade na gestão da dor realizadas por enfermeiros foram classificadas em organização das unidades e monitoramento do paciente e quanto ao período pré e pós-operatório, trazendo ações importantes, tais como definição de protocolos, capacitação da equipe, uso de instrumentos de avaliação da dor, intervenções não farmacológicas e avaliação por meio de auditorias, o que pode auxiliar os líderes e profissionais da prática no cuidado ao paciente.

Concluindo, esta revisão de pesquisa fornece uma visão abrangente das melhores práticas de enfermagem no manejo da dor pós-operatória em pacientes ortopédicos. Os resultados e recomendações obtidos podem constituir uma base sólida para melhorar as práticas de tratamento, melhorar os resultados dos pacientes e promover o desenvolvimento contínuo do tratamento neste grupo especial.
